# Morphology Control and Spectral Study of the 2D and Hierarchical Nanostructures Self-Assembled by the Chiral Alanine-Decorated Perylene Bisimides

**DOI:** 10.3390/molecules29194610

**Published:** 2024-09-28

**Authors:** Rui Qi, Xiaotian Huang, Ting Yang, Peng Luo, Wensheng Qi, Yin Zhang, Haimei Yuan, Hongmei Li, Jian Wang, Baohua Liu, Songzhi Xie

**Affiliations:** College of Food and Biological Engineering, Chengdu University, Chengdu 610106, China; qirui@cdu.edu.cn (R.Q.); 18756362123@163.com (X.H.); 2014244417@zmu.edu.cn (T.Y.); dejavu05@163.com (P.L.); qiwensheng@cdu.edu.cn (W.Q.); zhangyin@cdu.edu.cn (Y.Z.); m13880722953@163.com (H.Y.); lihongmei@cdu.edu.cn (H.L.); wangjian@cdu.edu.cn (J.W.); liubaohua@cdu.edu.cn (B.L.)

**Keywords:** nanostructures, π-conjugated molecules, morphologies, optical properties, molecular packing, spectral overlap

## Abstract

Tailoring the morphologies and optical properties of the 2D and hierarchical nanostructures self-assembled by the π-conjugated molecules is both interesting and challenging. Herein, a series of 2D ribbon-like nanostructures with single or multiple H-aggregated perylene bisimides (PBI) monolayer and hierarchical nanostructures (including straw-like, dumbbell-shaped, and rod-like nanostructures) are fabricated by solution self-assembly of three chiral alanine-decorated PBI. The influence of the solvent’s dissolving capacity, the chirality of alanine, and the preparation methods on the morphologies and optical properties of the nanostructures were extensively studied. It was observed that the hierarchical nanostructures are formed by the reorganization of the 2D ribbon-like nanostructures. The size of the 2D ribbon-like nanostructures and the amount of the hierarchical nanostructures increase with the decrease in the solvent’s dissolving capacity. The small chiral alanine moiety is unable to induce chirality in the nanostructures, owing to its low steric hindrance and the dominant strong π-π stacking interaction of the PBI skeleton. A weaker π-π stacking interaction and better H-aggregated arrangement of the PBI skeleton could reduce the low-wavelength fluorescence intensity. The process of heating, cooling, and aging promotes the formation of H-aggregation in the PBI skeleton. The region of spectral overlap of the PBI solutions increases with the decrease in the dissolving capacity of the solvent and the steric hindrance of the chiral alanine. This study supplies a view to tailor the morphologies and optical properties of the nanostructures, which could be used as sensors and photocatalysts.

## 1. Introduction

Nanomaterials fabricated by the π-conjugated molecules are attracting great interest because of their wide applications in sensors [1,2,3], bioimaging [4,5,6], catalysts [7,8,9], solar cells [10,11,12,13,14] and organic field-effect transistors [15,16,17,18], etc. In many cases, the properties of the nanomaterials are demonstrated to be highly dependent on their morphologies [19,20,21,22,23]. Thus, many efforts were tried to control the morphologies of the nanostructures [24,25]. Solution self-assembly was proved to be a low cost and effective method for preparing well-defined nanostructures. By means of the solution self-assembly of π-conjugated molecules, numerous well-defined nanostructures were obtained, such as spherical micelles [26,27,28,29], vesicles [25,30,31,32], fibers [33,34,35], ribbon-like micelles [36,37,38], and rectangular micelles [39,40,41], etc. In π-conjugated molecules, the π-π stacking interaction and molecular packing of the π-conjugated segments always play key roles in tailoring the sophisticated structures of the nanostructures, particularly during the fabrication of hierarchical nanostructures [39,42,43]. The factors affecting the π-π stacking interaction and molecular packing of the π-conjugated segments would strictly influence the morphologies and the properties of the nanostructures. For example, based on the self-assembly of the homopolymer and block copolymer of poly (3-hexylthiophene), Professor Park prepared the nanofibers, bundled nanofibers, and branched nanofibers by varying the solvent quality, polymer lengths, and block copolymer/homopolymer ratio [44]. However, most of the research focuses on the self-assembly of the amphiphilic π-conjugated molecules with good solubility. Control over the sophisticated structures of the nanostructures fabricated by the π-conjugated molecules with poor solubility is still a challenge.

Perylene bisimides belong to a class of small π-conjugated molecules that exhibit excellent photoelectric properties [45,46,47,48,49,50,51]. The π-π stacking interaction of the perylene skeleton and the solubility of the PBI are highly dependent on the chemical structures and location of the substituent groups. The substituent groups located on the perylene skeleton could strictly decrease the π-π stacking interaction and increase the solubility of the perylene skeleton, but destroy the parallel arrangement of the perylene skeleton, during the formation of the nanostructures. Contrarily, the substituent groups located on the imide sides could supply a chance to adjust the π-π stacking interaction and solubility of the perylene skeleton without obviously changing its parallel arrangement. Recently, the self-assembly of the PBI with good solubility was much studied, and a series of nanofibers and nanoribbons were obtained. However, the self-assembly of the PBI with poor solubility is still a challenge. Up to date, few researchers focus on tailoring the sophisticated structures of the nanostructures self-assembled by the PBI with poor solubility.

In our previous paper [38], we studied the self-assembly of two chiral PBI, namely PBI-D-14 and PBI-L-164, which contain the small chiral groups (D-alanine or L-alanine) and triethylene glycol monomethyl. The shapes, sizes, and heights of the obtained 2D nanostructures could be tailored by the chirality of alanine and the dissolving capacity of the solvent. However, the optical properties of those nanostructures were only obviously influenced by the type of the solvent. The chirality of alanine had little impact on the optical properties of those nanostructures, due to the little difference in steric resistance between D-alanine and L-alanine. Herein, we further studied the self-assembly and the optical properties of three chiral alanine-decorated PBI with reduced solubility. These chiral alanine-decorated PBI (PBI-L, PBI-D, and PBI-β) were synthesized by the L-alanine (D-alanine or β-alanine) and the 3,4,9,10-perylene tetracarboxylic dianhydride (PTCDA). The morphologies and optical properties of the nanostructures were highly dependent on the solvent, the chirality of alanine, and preparation methods which mainly affected the π-π stacking interaction and molecular packing of the perylene skeleton. Through a heating, cooling, and aging process, a series of 2D nanostructures and hierarchical nanostructures (such as straw-like, dumbbell-like, and rod-like nanostructures) were controllably fabricated by the self-assembly of the chiral alanine-decorated PBI in alcoholic solvents. These hierarchical nanostructures were demonstrated to be formed by the reorganization of the 2D ribbon-like nanostructures, induced by the large interface energy between the surface of the 2D ribbon-like micelles and the solvent. The size of the 2D ribbon-like nanostructures and the amount of the hierarchical nanostructures increased with the decrease in the dissolving capacity of the solvent. Due to the different steric hindrance of the chiral alanine, the PBI-L and PBI-D self-assembled into the 2D ribbon-like nanostructures with a single H-aggregated PBI monolayer and multiple H-aggregated PBI monolayers, respectively. Meanwhile, the PBI-β could only self-assemble into well-defined 2D ribbon-like nanostructures in methanol. In aqueous solutions, the sophisticated structures of the 2D nanostructures formed by the ionization–neutralization method were different from those formed by a heating, cooling, and aging process, which was conducive to the formation of H-aggregation of the PBI skeleton. As the variation in the dissolving capacity of the solvent took place, the UV-Vis absorption spectra of PBI-L, and PBI-D, changed more significantly than those of PBI-β due to the different steric hindrance of the chiral alanine on the PBI skeleton. The emission spectra of the three chiral alanine-decorated PBI decreased around 618.7 nm and increased between 460.0 and 520.0 nm, respectively, with the decrease in the dissolving capacity of the alcoholic solvent and the steric hindrance of the chiral alanine. This was attributed to the decrease in the crystalline structures formed by the H-aggregation of PBI skeleton. The region of spectral overlap of the PBI systems increased with the decrease in the dissolving capacity of the solvent and the steric hindrance of the chiral alanine. Notably, the region of spectral overlap of the nanostructures formed by the ionization–neutralization method was larger than that of the nanostructures formed by the heating, cooling, and aging process. This study supplies a view to tailor the morphologies and optical properties of the nanostructures which could be used as sensors and photocatalysis.

## 2. Results and Discussions

### 2.1. Synthesis and Characterization of the Alanine-Decorated PBI

**Scheme 1:**
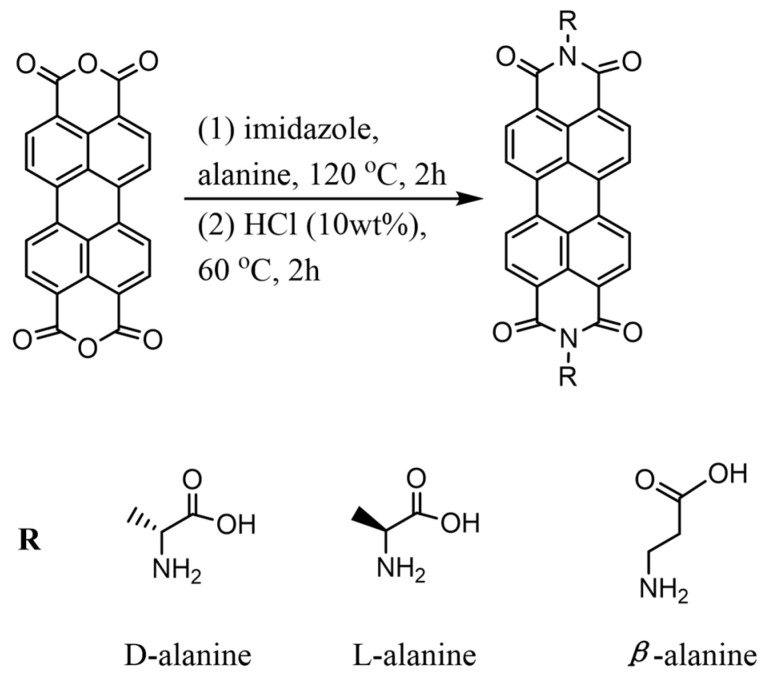
The synthetic processes of the three alanine-decorated PBI.

The synthetic processes of the three alanine-decorated PBI were descripted in detail in supporting information, as shown in Scheme 1. The nuclear magnetic resonance spectroscopy (^1^H NMR) was first utilized to confirm the chemical structures of the three alanine-decorated PBI (Supplementary Materials: Figures S1–S3). The chemical shifts at 7.31–7.80 ppm are contributed from the protons of the aromatic structure of the perylene skeleton. The signals at 5.30 ppm and 1.67 ppm are derived from the protons of methylene and methyl groups of D-alanine and L-alanine, respectively. The signals at 4.05 ppm and 2.47 ppm are derived from the protons of methylene groups of β-alanine. The ratios of the perylene group and alanine were calculated to be 1:2 by the ^1^H NMR results. The ^1^H NMR results prove the successful synthesis of the objective structures. The molecular weights (M) of all the three alanine-decorated PBI were found to be 536 *m*/*z* by the matrix-assisted laser desorption ionization time-of-flight mass spectrometry (MALDI-TOF MS) (Figures S4–S6), which are corresponding with their theoretical values (M = 536 *m*/*z*). The formation of the three alanine-decorated PBI was also confirmed by the fourier transform infrared spectrometer (FT-IR). Figures S7–S9 show that the N-H bending vibration of alanine at about 1620.3 and 1596.5 cm^−1^ and the C=O stretching vibration of PTCDA at about 1774.5 cm^−1^ disappeared, and the C=O stretching vibration of amide group at 1696.1 and 1653.7 cm^−1^ newly appeared. The results of the ^1^H NMR, MALDI-TOF MS, and FT-IR confirm the successful synthesis of the three alanine-decorated PBI.

### 2.2. Self-Assembly of the PBI-L in i-PrOH

**Figure 1:**
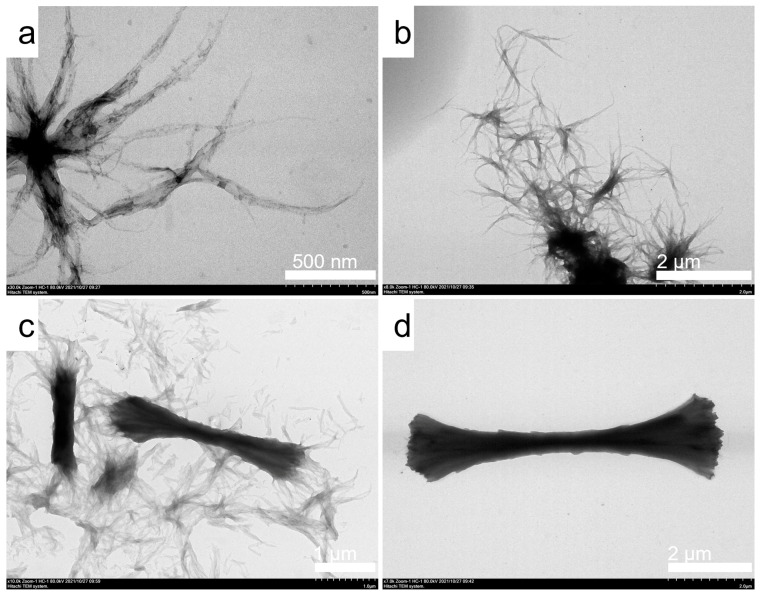
The TEM images of the nanostructures formed by the PBI-L in *i*-PrOH (c = 0.0001 mg·mL^−1^) after a heating, cooling, and aging process, (**a**) the 2D nanostructures, (**b**) the straw-like nanostructures, (**c**) the immature dumbbell-shaped nanostructures, (**d**) the dumbbell-shaped nanostructures.

**Figure 2:**
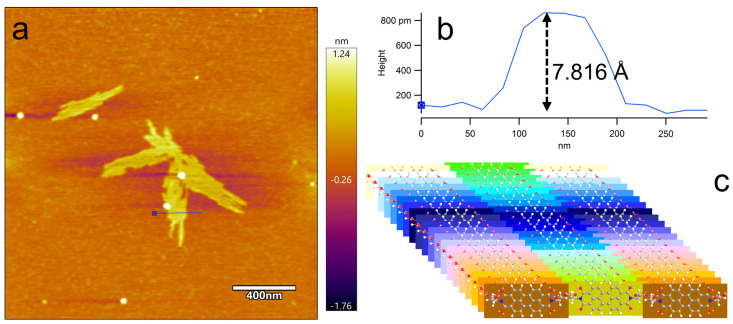
The AFM images of the 2D ribbon-like nanostructures formed by the PBI-L in *i*-PrOH (c = 0.0001 mg·mL^−1^) after a heating, cooling, and aging process, (**a**) the height image, (**b**) the height of the nanostructures, and (**c**) the probable molecular packing of the PBI-L in the nanostructures.

**Figure 3:**
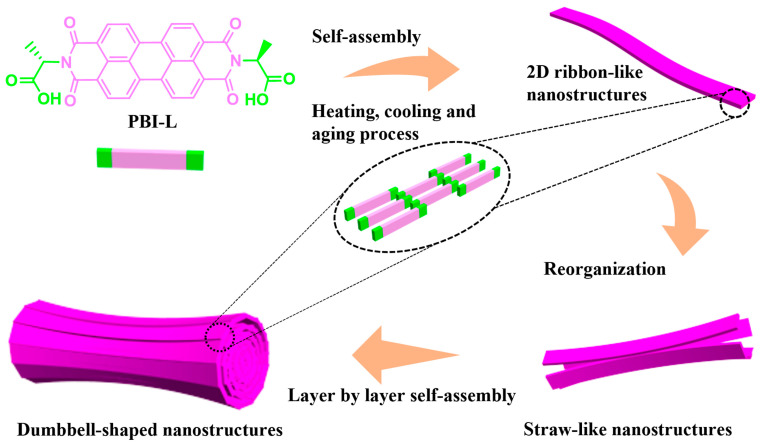
The probable formation process of the 2D ribbon-like nanostructures, straw-like nanostructures, and dumbbell-shaped nanostructures formed by the PBI-L in *i*-PrOH (c = 0.0001 mg·mL^−1^) after a heating, cooling, and aging process.

**Figure 4:**
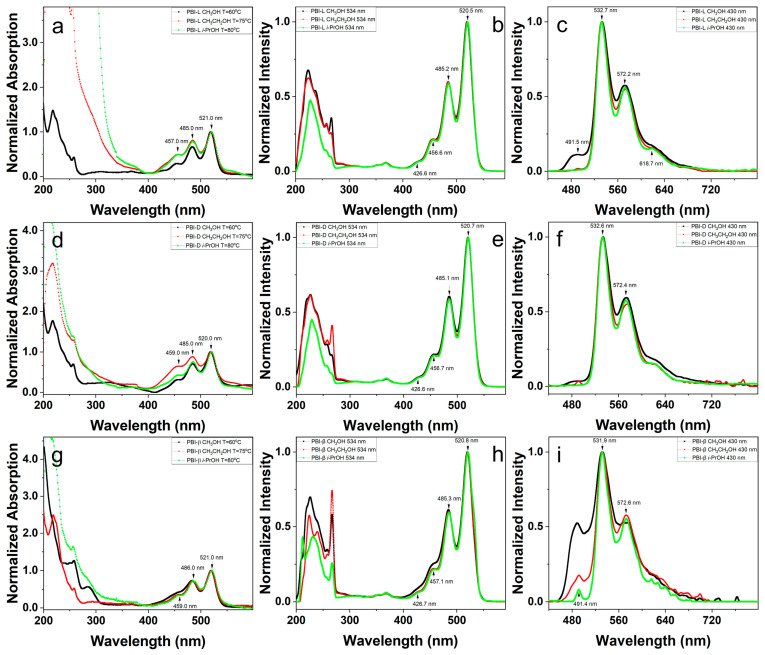
The UV-Vis absorption spectra (**a**,**d**,**g**), the fluorescence excitation spectra (at an emission band of 534.0 nm) (**b**,**e**,**h**), and the fluorescence emission spectra (excited by 430.0 nm) (**c**,**f**,**i**) of the PBI-L (**a**–**c**), PBI-D (**d**–**f**), and PBI-β (**g**–**i**) in *i*-PrOH, ethanol and methanol, respectively (c = 0.0001 mg·mL^−1^), after undergoing a heating, cooling, and aging process.

Because of the strong π-π stacking interaction of perylene skeleton and hydrogen bonding of the carboxyl groups, the solubility of the alanine-decorated PBI is very poor. To obtain well-defined nanostructures, we studied the self-assembly of the alanine-decorated PBI in low-concentration solutions. The transmission electron microscopy (TEM, Hitachi HT7800), the high-resolution transmission electron microscope (HRTEM, JEM-F200), the selected area electron diffraction (SAED, JEM-F200), and the atomic force microscopy (AFM, Bruker Dimension Icon) were utilized to characterize the sophisticated structures of the nanostructures. The self-assembly of PBI-L in *i*-PrOH (c = 0.0001 mg·mL^−1^) was first studied. After being heated at 80 °C for 2 h, the solution was slowly cooled down to room temperature and then aged for 24 h at room temperature. During the heating, cooling, and aging process, a series of 2D ribbon-like micelles, straw-like nanostructures, and dumbbell-shaped nanostructures were found in the solution of PBI-L in *i*-PrOH (shown in Figure 1 and Figure S10). It is easy to find that the straw-like nanostructures were composed of a series of 2D ribbon-like micelles. Notably, many immature dumbbell-shaped nanostructures were also found in this solution. Figure 1c and Figure S10c show that the ends of these immature dumbbell-shaped nanostructures were composed of a series of 2D ribbon-like micelles. Thus, it is reasonable to conclude that the dumbbell-shaped nanostructures should be formed as a result of the reorganization of the 2D ribbon-like micelles. The straw-like nanostructures were probably the precursors for the formation of the dumbbell-shaped nanostructures. The formations of the 2D ribbon-like micelles and dumbbell-shaped nanostructures were also be confirmed by the HRTEM results (Figure S11a,b). Additionally, no obvious crystal diffraction peaks were observed in the SAED results (Figure S11c,d), potentially attributable to the weak crystallinity of PBI-L or the extremely thin nature of the 2D nanostructures. Table S1 shows that the number-average length (L_n_) of the dumbbell-shaped nanostructures was calculated to be 3039 nm. The number-average diameter (D_1,n_) of the ends and the D_2,n_ of the central parts of the dumbbell-shaped nanostructures were calculated to be 1071 and 358 nm, respectively. The values of the L_w_/L_n_ (1.33, L_w_ is the weight-average length of the dumbbell-shaped nanostructures) and the D_1,w_/D_1,n_ (1.33, D_w_ is the weight-average diameter of the dumbbell-shaped nanostructures) indicate broad size distributions. This broad dispersity is also confirmed by the statistical size distributions. Figure S12a,b shows that the statistical lengths of the dumbbell-shaped nanostructures and the statistical diameters of the ends of the dumbbell-shaped nanostructures range from 900 to 7500 nm, and from 200 to 2700 nm, respectively. The dispersity of the diameters of the central parts of the dumbbell-shaped nanostructures was narrow, which was confirmed by the value of the D_2,w_/D_2,n_ (1.07) and the corresponding statistical size distribution (shown in Figure S12c). The AFM results (Figure 2a,b and Figure S13a,b) show that the heights of the 2D ribbon-like micelles were probably between 7.816 and 8.601 Å, which were a little larger than the estimated width of the PBI skeleton (Figure S14). This means that the 2D ribbon-like micelles were composed by a laminar PBI monolayer (shown in Figure 2c). Therefore, a possible process (Figure 3) of the formation of these 2D nanostructures was proposed, as follows: After being heated at 80 °C for 2 h, the PBI-L was dissolved in *i*-PrOH. During the cooling and aging process, the solubility of the PBI-L decreased, the PBI-L became aggregate and self-assembled into the 2D ribbon-like micelles composed of a laminar PBI monolayer, driven by the strong π-π interaction of the perylene skeleton, intermolecular van der Waals force, and the hydrogen bonding of the carboxyl groups. At the same time, the formed 2D ribbon-like micelles further reorganized, layer by layer, into straw-like nanostructures and dumbbell-shaped nanostructures, due to the large interface energy between the surface of the 2D ribbon-like micelles and the solvent. Figure 4 shows the UV-Vis absorption spectra, the fluorescence excitation spectra, and the fluorescence emission spectra of the assembly system of PBI-L in *i*-PrOH. There are three absorption peaks around 521.0, 485.0 and 457.0 nm between 400.0 and 550.0 nm, corresponding to the S_0-0_, S_0-1_ and S_0-2_ vibronic transitions of the perylene skeleton, respectively. The fluorescence excitation spectra (Figure 4b and Figure S15a) show that the excitation peaks for the emissions at 534.0 and 574.0 nm between 400.0 and 550.0 nm are similar to the UV-Vis absorption peaks. The relative intensity ratios of the emission at 534.0 nm excited by 485.2 and 456.6 nm are nearly the same as the corresponding values of the emission at 574.0 nm, and smaller than the relative absorption ratios at 485.0 and 457.0 nm. The fluorescence emission spectra (Figure 4c and Figure S15b) show that there are three emission peaks around 532.3, 572.3, and 618.7 nm excited by 430.0 and 450.0 nm. The relative fluorescence emission ratio of 572.3 nm excited by 430.0 nm is slightly larger than the corresponding value excited by 450.0 nm. The appearance of the emission peak around 618.7 nm was probably due to the formation of the crystal structures of the perylene skeleton [38,52].

### 2.3. Effect of the Solvent on the Self-Assembly of the PBI-L

**Figure 5:**
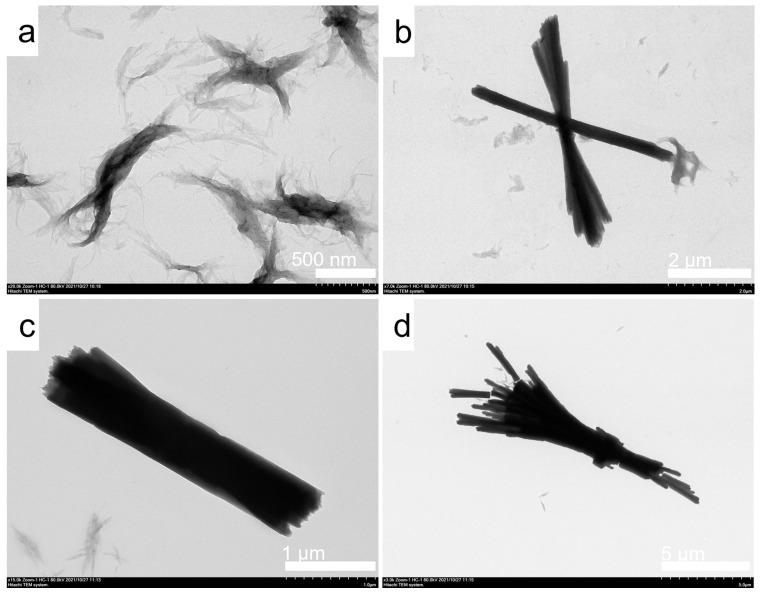
The TEM images of the nanostructures formed by the PBI-L in ethanol (**a**,**b**) and methanol (**c**,**d**) (c = 0.0001 mg·mL^−1^) after a heating, cooling, and aging process.

In our previous study, the shapes and sizes of the nanostructures formed by the PBI were proved to be influenced by the dissolving capacity of the alcoholic solvents [38]. Thus, the methanol and ethanol were selected as the other solvents for the self-assembly of the PBI-L. Figure 5a,b and Figure S16a,b show that a series of ultrathin 2D ribbon-like micelles, straw-like nanostructures, and rod-like nanostructures were found in ethanol after a heating, cooling, and aging process. The straw-like nanostructures and rod-like nanostructures were the dominate structures. These straw-like nanostructures and rod-like nanostructures in ethanol should be formed by the reorganization of the ultrathin 2D micelles, similar to the hierarchical nanostructures found in *i*-PrOH. The L_1,n_ of the straw-like nanostructures was calculated to be 1258 nm. The L_1,w_/L_1,n_ (1.07) of the straw-like nanostructures indicates a good size distribution, which was also confirmed by the statistical length distribution. Figure S17a shows the length of the straw-like nanostructures ranged from 200 to 2700 nm. The L_2,n_ of the rod-like nanostructures was about 3745 nm, which is larger than the corresponding value of the dumbbell-shaped nanostructures in *i*-PrOH. The statistical length distribution ranging from 1800 to 6400 nm (shown in Figure S17b) and the L_2,w_/L_2,n_ (1.08) of the rod-like nanostructures prove a narrow length distribution. The D_2,n_ of the central parts of the rod-like nanostructures was about 268 nm. The statistical diameter distribution ranging from 100 to 550 nm (shown in Figure S17c) and the D_2,w_/D_2,n_ (1.24) of the rod-like nanostructures proves a broad size distribution. In methanol, the PBI-L mainly self-assembled into rod-like nanostructures and large complicated nanostructures. Only a small number of ultrathin 2D micelles and straw-like nanostructures were found (shown in Figure 5c,d and Figure S16c,d). As a corollary, the rod-like nanostructures in methanol should be formed by the reorganization of the ultrathin 2D micelles. Meanwhile, the formation of the large complicated nanostructures was due to the aggregation of the rod-like nanostructures.

The above results show that the number of the ultrathin 2D micelles and hierarchical nanostructures decreased and increased, respectively, with the decrease in the dissolving capacity of the solvent. This could be explained as follows: As the dissolving capacity of the solvent decreased, the π-π interaction of the perylene skeleton and the interface energy between the surface of the 2D ribbon-like micelles and the solvent both increased, which induced more 2D micelles further to aggregate to form hierarchical nanostructures.

Figure 4a shows that the UV-Vis absorption peaks of the assembly systems of PBI-L, located at about 521.0, 485.0, and 457.0 nm, slightly blue-shifted with the increase in the solvent polarity. This shift indicates that an increase in solvent polarity elevates the excitation energy of the PBI skeleton. The relative absorption ratios around 485.0 and 457.0 nm for the methanol solution are significantly lower than the corresponding values for the *i*-PrOH and ethanol solutions (shown in Table S2). The fluorescence excitation spectra (Figure 4b and Figure S15a and Table S3) for the emissions at 534.0 and 574.0 nm of the three systems between 400.0 and 550.0 nm are almost the same. The fluorescence emission spectra are shown in Figure 4c and Figure S15b. As the solvent polarity increased, the fluorescence emission peaks around 532.3 nm, excited by 430.0 nm, were slightly red-shifted. Meanwhile, the fluorescence emission peaks around 572.3 nm, excited by 430.0 nm, first increased and then decreased (shown in Table S4). The fluorescence emission ratios around 572.3 nm, excited by 430.0 nm, first decreased and then increased with the increase in the solvent polarity. Figure S15b and Table S4 show that the fluorescence emission peaks around 531.3 and 572.4 nm, excited by 450.0 nm, all slightly red-shifted with the increase in the solvent polarity. The fluorescence emission ratios around 572.6 nm, excited by 450.0 nm, also slightly increased with the increase in the solvent polarity. Meanwhile, the emission peak of the crystal structures around 618.7 nm diminished with the increase in the solvent polarity; this is because the solubility of the PBI-L decreased more quickly in a stronger polar solvent during the cooling process. There was not enough time for the PBI-L in methanol to be arranged regularly to form the crystal structures [37,38]. Notably, a new broad emission band around 491.5 nm was observed in the methanol solution when excited by 430.0 nm. On the contrary, no obvious emission band around 491.5 nm was observed in methanol solution when excited by 450.0 nm. This means that the emission band around 491.5 nm was mainly contributed by the UV-Vis absorption at 430.0 nm. Meanwhile, the emission band around 491.5 nm disappeared with the decrease in the solvent polarity, which was probably due to the increase in crystal structures.

### 2.4. Effect of the Chirality on the Self-Assembly of the PBI

**Figure 6:**
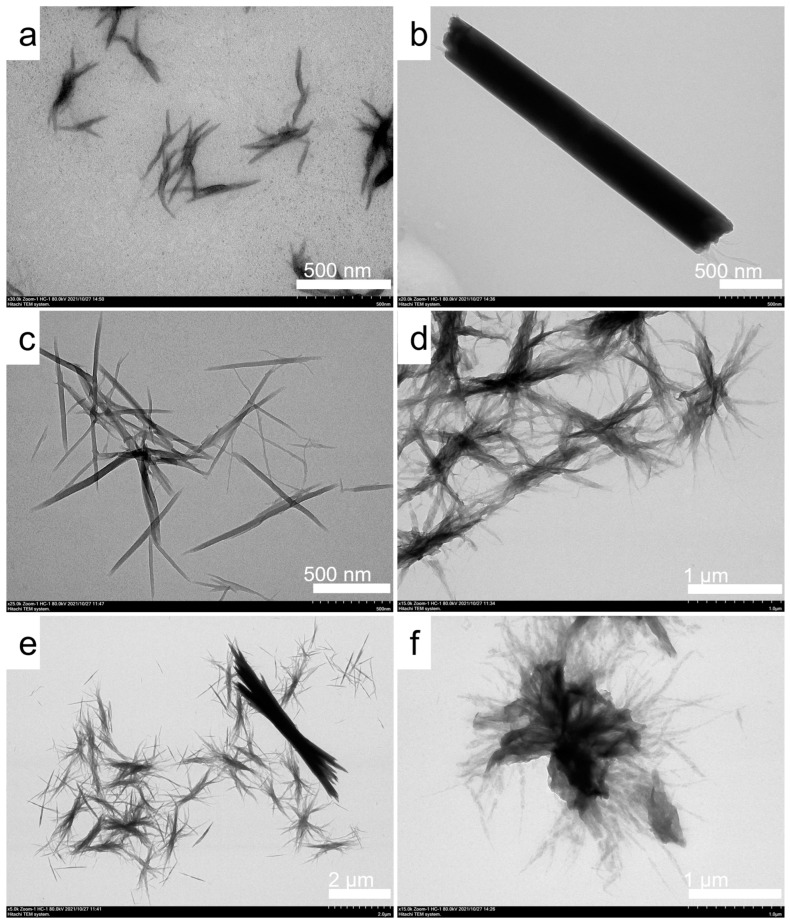
The TEM images of the nanostructures formed by the PBI-D in *i*-PrOH (**a**,**b**), ethanol (**c**–**e**) and methanol (**f**) (c = 0.0001 mg·mL^−1^) after a heating, cooling, and aging process.

The conformation and stacking pattern of the perylene skeleton were proved to be influenced by the substituent groups of the PBI, which further affected the π-π interaction of the perylene skeleton and the morphologies of the nanostructures. Herein, D-alanine and β-alanine-decorated PBI were chosen to investigate the effect of the chirality of the small groups linked to the imide group on the morphologies and optical properties of the nanostructures. In *i*-PrOH, the PBI-D self-assembled into the regular 2D ribbon-like nanostructures (Figure 6a and Figure S18a) and rod-like nanostructures (Figure 6b and Figure S18b). The regular 2D ribbon-like nanostructures were the dominant structures. In ethanol, the PBI-D self-assembled into the regular 2D ribbon-like nanostructures, straw-like nanostructures, and rod-like nanostructures (Figure 6c–e and Figure S18c,d). The 2D ribbon-like nanostructures and straw-like nanostructures were the dominant structures. In methanol, the PBI-D mainly self-assembled into straw-like nanostructures (Figure 6f and Figure S18e,f). Obviously, the L_n_ (725 nm) and the number average width (W_n_ = 35 nm) of the 2D ribbon-like nanostructures formed in ethanol are larger than the corresponding sizes (L_n_ = 253 nm, W_n_ = 27 nm) of the 2D ribbon-like nanostructures formed in *i*-PrOH. The values of the L_w_/L_n_ and W_w_/W_n_ (W_w_ is the weight average width) of the 2D ribbon-like nanostructures formed in *i*-PrOH and ethanol are shown in Table S1, which demonstrates the good size distributions. The corresponding statistical size distributions are shown in Figure S19. The D_n_ of the straw-like nanostructures formed in methanol was calculated to be 2100 nm. The D_w_/D_n_ (1.13) and the statistical size distribution (shown in Figure S19e) also indicate a good size distribution.

**Figure 7:**
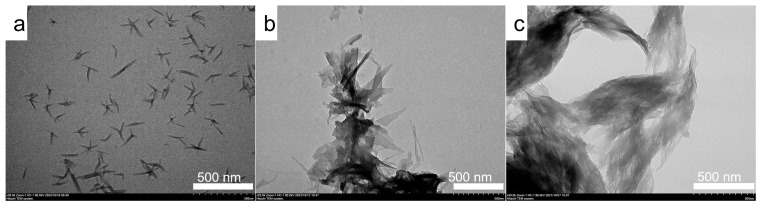
The TEM images of the nanostructures formed by the PBI-β in *i*-PrOH (**a**), ethanol (**b**), and methanol (**c**) (c = 0.0001 mg·mL^−1^) after a heating, cooling, and aging process.

In *i*-PrOH, the PBI-β self-assembled into a series of regular 2D ribbon-like nanostructures (shown in Figure 7a and Figure S20a,b). No hierarchical nanostructures were founded. The L_n_ and W_n_ of the 2D ribbon-like nanostructures were calculated to be 146 and 21 nm, respectively. The L_w_/L_n_ (1.10) and W_w_/W_n_ (1.09) of the 2D ribbon-like nanostructures indicate the narrow size distributions. The statistical size distributions were shown in Figure S21a,b. On the contrary, hierarchical nanostructures formed by the aggregation of the 2D ribbon-like nanostructures (shown in Figure 7b and Figure S20c,d) were found in ethanol of the PBI-β. Only a few individual 2D ribbon-like nanostructures could be observed. The length (L_n_ = 368 nm) and width of the 2D ribbon-like nanostructures formed in ethanol were obviously larger than the corresponding sizes of the regular 2D ribbon-like nanostructures formed in *i*-PrOH. The L_w_/L_n_ of the 2D ribbon-like nanostructures was calculated to be 1.36, implying a broad length distribution. The statistical length distribution is shown Figure S21c. In methanol, the PBI-β self-assembled into spindle-like nanostructures composed of a series of ultrathin 2D micelles (Figure 7c and Figure S20e,f). The L_n_ of the spindle-like nanostructures was calculated to be 1009 nm. The L_w_/L_n_ (1.07) of the spindle-like nanostructures and the statistical size distribution (shown in Figure S21d) indicate a good size distribution.

The above results demonstrate that the morphologies of the nanostructures were obviously affected by the chirality of the small groups linked to the imide group. The 2D ribbon-like nanostructures formed by PBI-β in *i*-PrOH and PBI-D in *i*-PrOH and ethanol were more regular than the 2D ribbon-like micelles formed by PBI-L in *i*-PrOH. The electron density of the former was dark and uneven, which implies that the former should be composed of several ultrathin 2D micelles (shown in Figure S22). The amount of the hierarchical nanostructures in the assembly systems of PBI-D and PBI-β also increased with the decrease in the dissolving capacity of the solvent. Notably, no chiral nanostructures were found in all of the assembly systems. It is probably because of this that the steric hindrance of the small groups linked to the imide group was too weak to remarkably affect the arrangement of the perylene skeleton during the formation of the nanostructures. However, the π-π stacking interaction of the perylene skeleton decreased as the steric hindrance of the small groups linked to the imide group increased. The magnitude of this π-π stacking interaction in the perylene skeleton is negatively correlated with its solubility. In fact, the solubility of the PBI-D in alcoholic solutions was found to be the best, and the solubility of the PBI-β was the worst. Therefore, the differences in the nanostructures formed by the three alanine-decorated PBI can be attributed to the variation in the π-π stacking interaction and solubility of the perylene skeleton. During the cooling and aging process, there was more time for the PBI-D to adjust the arrangement of the perylene skeleton due to the weaker π-π stacking interaction of the perylene skeleton. Thus, the 2D ribbon-like nanostructures were more regular and the amount of the hierarchical nanostructures was relatively fewer in the assembly systems of the PBI-D. In the systems of PBI-β, the hierarchical nanostructures were more irregular and complex, owing to the stronger π-π stacking interaction of the perylene skeleton and the enhanced interface energy between the surface of the 2D ribbon-like nanostructures and the solvent. The amounts of the hierarchical nanostructures in the systems of the three alanine-decorated PBI all increase with the decrease in the dissolving capacity of the solvent.

The optical properties of the nanostructures were also found to be obviously influenced by the chirality of the small groups linked to the imide group and solvent polarity. There are also three UV-Vis absorption peaks (Figure 4d) for the PBI-D in *i*-PrOH, ethanol, and methanol between 400.0 and 550.0 nm. The variations in the absorption peaks around 521.0 and 485.0 nm with the increase in the solvent polarity were similar with those of the systems of the PBI-L. Meanwhile, the absorption peak around 456.0 nm first increased and then decreased with the increase in the solvent polarity. The absorption ratios around 485.0 and 459.0 nm for the PBI-D in ethanol and methanol were much larger than the corresponding values for the PBI-L in ethanol and methanol. However, the absorption ratios around 485.0 and 459.0 nm for the PBI-D in *i*-PrOH were much smaller than the corresponding values for the PBI-L in *i*-PrOH. The fluorescence excitation spectra (Figure 4e and Figure S15c) for the emissions at 534.0 and 574.0 nm of the PBI-D in the three solutions between 400.0 and 550.0 nm are almost the same. The variation in the fluorescence emission spectra (Figure 4f and Figure S15d) of the PBI-D solutions with the increase in the solvent polarity was similar to that of the PBI-L solutions. However, the degree of the variation in the former was larger than the latter. The emission peaks of the crystal structures around 618.7 nm, excited by 430.0 and 450.0 nm, also diminished with the increase in the solvent polarity. The fluorescence emission ratios around 491.3 nm for the PBI-D systems were much smaller than the corresponding values for the PBI-L systems.

Figure 4g shows that the UV-Vis absorption peaks of the PBI-β around 521.0 and 486.0 nm slightly blue-shifted with the increase in the solvent polarity. The relative absorption ratios around 486.0 nm changed slightly with the variation in the solvent polarity. The excitation peaks (Figure 4h and Figure S15e) of the PBI-β for the emissions at 534.0 and 574.0 nm, located around 520.8, 485.3, and 457.1 nm, all blue-shifted with the increase in the solvent polarity. The relative emission ratios of the PBI-β at 534.0 and 574.0 nm, excited by 485.3 and 457.1 nm, increased with the increase in the solvent polarity. The fluorescence emission spectra (Figure 4i and Figure S15f) of the PBI-β were much different from those of the PBI-L and PBI-D. The fluorescence emission peaks of the PBI-β around 531.9 nm, excited by 430.0 and 450.0 nm, remained unchanged with the variation in the solvent polarity. The fluorescence emission peaks of the PBI-β around 568.6 nm, excited by 430.0 and 450.0 nm in methanol, obviously blue-shifted. Notably, the fluorescence emission peaks of the PBI-β around 491.4 nm, excited by 430.0 and 450.0 nm, were found in all of the three solutions. This may be due to the decrease in the crystal structures of the PBI-β systems. In fact, no significant emission peaks around 618.7 nm were found in the three PBI-β solutions. The relative emission ratios of 491.4 nm decreased with the decrease in the solvent polarity. The relative emission ratios at 491.4 nm, excited by 430.0 nm, were much larger than the corresponding values excited by 450.0 nm.

In the previous study [38], the shapes of the nanostructures formed by PBI-L-164 and PBI-D-164 were similar, and no hierarchical nanostructures were observed. The optical properties of the assembly systems exhibited only slight changes with variations in chirality and the dissolving capacity of the alcoholic solvents. However, in this study, the shapes of the nanostructures formed by PBI-L and PBI-D were significantly influenced by both chirality and the dissolving capacity of the alcoholic solvents, whereas the optical properties of the assembly systems of PBI-L and PBI-D were only slightly affected by these factors. The similar variation in the optical properties observed in the two studies can probably be attributed to the fact that the steric hindrance of the side groups containing L-alanine is similar to that of the side groups containing D-alanine, leading to similar π-π stacking interactions and arrangements of the perylene skeleton. The different variation in the morphologies of the nanostructures observed in the two studies could be explained as follows: For PBI-L-164 and PBI-D-164, the steric hindrance of the L-alanine and D-alanine in the side groups is much smaller than that of the triethylene glycol monomethyl segment. This leads to more similar π-π stacking interactions and arrangements of the perylene skeleton, resulting in nanostructures whose shapes are slightly influenced by the chirality of the alanine. Additionally, the solubility of PBI in PBI-L-164 and PBI-D-164 is enhanced by the soluble triethylene glycol monomethyl segment. This enhancement leads to smaller π-π stacking interactions, reduced intermolecular van der Waals force, and lower interface energy between the surface of the nanostructures and the solvent, making it unsuitable for forming hierarchical nanostructures. Similarly, due to significantly different steric hindrance, the shapes and optical properties of the nanostructures formed by PBI-β in this study differ greatly from those formed by PBI-L and PBI-D. These indicate that the chirality of the side segments and the solubility of the perylene skeleton have a significant influence on the morphologies and optical properties of the nanostructures. In the two studies, the influence of the chiral moieties on the morphologies and optical properties of the nanostructures is fundamentally determined by steric hindrance.

### 2.5. Self-Assembly of the Chiral PBI in Aqueous Solutions

The effect of different methods on the self-assembly of the three PBI in deionized water was studied. First, the solutions of the three PBI were all heated at 80 °C for 2 h, and then slowly cooled and aged for 24 h at the temperature. After this process, the PBI-L, PBI-D, and PBI-β all self-assembled into 2D ribbon-like micelles (Figure S23). The electron densities between the edge regions and central regions of the 2D ribbon-like micelles formed by PBI-L were different, which implies that the 2D ribbon-like micelles were composed of multiple thinner layers. The L_n_ and W_n_ of the 2D ribbon-like micelles formed by PBI-L were about 890 and 76 nm, respectively. The size distributions (Figure S24a,b) and the values of L_w_/L_n_ (1.20) and W_w_/W_n_ (1.09) indicate good size dispersity for the 2D ribbon-like micelles formed by PBI-L. The 2D ribbon-like micelles formed by PBI-D were also composed of multiple thinner layers. The width of the 2D ribbon-like micelles formed by PBI-D was similar to that of the 2D ribbon-like micelles formed by PBI-L. However, the length of the 2D ribbon-like micelles formed by PBI-D was obviously larger that of the 2D ribbon-like micelles formed by PBI-L. In the aqueous solution of PBI-β, most of the 2D ribbon-like micelles self-aggregated to form irregular aggregates. Additionally, some 2D ribbon-like micelles were apparently composed of multiple thinner layers. These may be caused by the strong π-π stacking interaction and the hydrogen bonding. The width of the 2D ribbon-like micelles formed by PBI-β ranged from 15 to 125 nm (Figure S24c), which was a little smaller than that of the 2D ribbon-like micelles formed by PBI-L. Secondly, the three PBI were first dissolved in deionized water with TEA, then a certain amount of hydrochloric acid was added. After the second process, the PBI-L, PBI-D, and PBI-β also self-assembled into 2D ribbon-like micelles (Figure S23e,g,h). The AFM results (Figure S25) indicate that these 2D ribbon-like nanostructures were all composed of multiple layers, as shown in Figure S22. The statistical size distributions of these 2D ribbon-like micelles were shown in Figure S26 and Table S1. The *L*_n_ values of the 2D ribbon-like nanostructures formed by PBI-L, PBI-D, and PBI-β increase progressively. The W_n_ values for the 2D ribbon-like nanostructures formed by PBI-L and PBI-D are significantly smaller than that observed for the 2D ribbon-like nanostructures formed by PBI-β. The larger sizes of the 2D ribbon-like nanostructures formed by PBI-β were probably attributed to the stronger π-π stacking interaction of the perylene skeleton, originating from the smaller steric hindrance of the β-alanine. Figure S27 shows that as nanostructures formed, the O-H stretching vibration of the COOH group in PBI decreased at approximately 3500.0 cm^−1^, the C=O stretching vibration of the COOH group in PBI-L shifted from 1748.9 cm^−1^ to 1743.8 cm^−1^, the C=O stretching vibration of the amides in PBI-L shifted from 1694.8 cm^−1^ to 1691.9 cm^−1^, the C=O stretching vibration of the amides in PBI-D shifted from 1653.7 cm^−1^ to 1640.9 cm^−1^, and the C=O stretching vibration of the amides in PBI-β shifted from 1653.0 cm^−1^ to 1639.4 cm^−1^. The FT-IR results confirm the presence of intermolecular hydrogen bonds within the nanostructures of PBI that were formed through an ionization–neutralization method. The molecular packing patterns in the PBI nanostructures formed by the ionization–neutralization method were also studied. The X-ray powder diffraction (XRD) results presented in Figure S28 confirm the presence of a unit cell exhibiting a two-dimensional (2D) crystal structure (Figure S29) within the nanostructures formed by PBI-L, PBI-D, and PBI-β. For PBI-L, the peaks at approximately 2θ = 26.62° and 2θ = 25.79° could be attributed to π-π stacking between the perylene planes with distances of 3.349 Å and 3.454 Å, respectively. The peak at 2θ = 16.15° corresponds to the height of the 2D laminar monolayers of PBI-L, measured to be 5.487 Å, and represents the height dimension of the unit cell. The peak at 2θ = 9.26° indicates a height that corresponds to twice the unit cell height, measured as 9.552 Å. The peak at 2θ = 6.42° corresponds to the length of the unit cell, which was 13.776 Å and slightly shorter than the length of PBI-L. For PBI-D, only one peak was observed at approximately 2θ = 25.79°, attributed to π-π stacking between the perylene planes with a distance of 3.454 Å. The other parameters of the unit cell for PBI-D in the nanostructures were similar to those of PBI-L. The unit cell of PBI-β was also comparable to those of PBI-L and PBI-D. The π-π stacking distance between the perylene planes of PBI-β was 3.447 Å, slightly smaller than the corresponding values for PBI-L and PBI-D, due to reduced steric hindrance. The unit cell parameters for the three PBI in nanostructures are shown in Table S5. The above results demonstrate that the morphologies of the nanostructures were highly dependent on the preparation methods.

**Figure 8:**
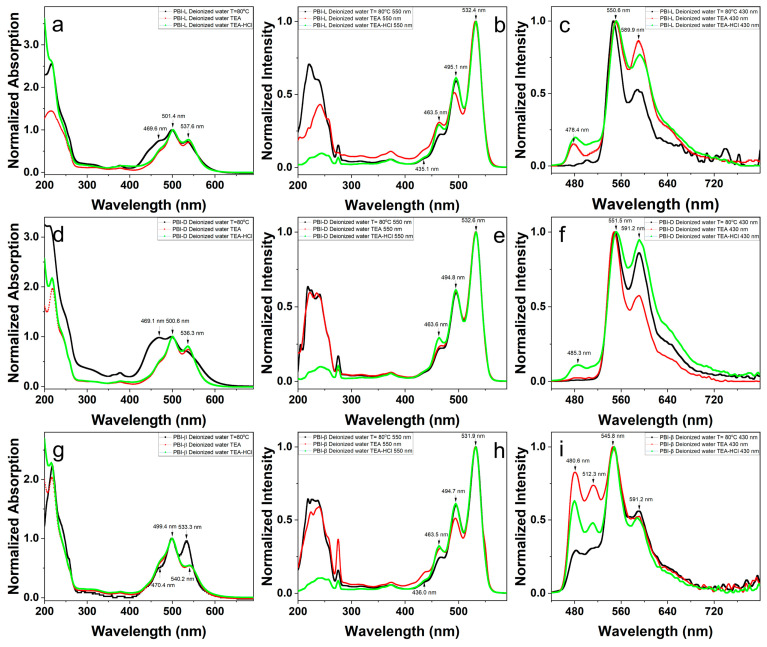
The UV-Vis absorption spectra (**a**,**d**,**g**), the fluorescence excitation spectra (at an emission band of 550.0 nm) (**b**,**e**,**h**) and the fluorescence emission spectra (excited by 430.0 nm) (**c**,**f**,**i**) of the PBI-L (**a**–**c**), PBI-D (**d**–**f**), and PBI-β (**g**–**i**) in aqueous solutions through different processes.

The optical properties of the nanostructures fabricated by the above two methods were studied in detail. Figure 8a shows that the UV-Vis spectra of PBI-L processed by different methods were significantly different. The pattern of the spectrum of the system of PBI-L with TEA and HCl was similar to that of the system of the dissolved PBI-L with TEA. However, the corresponding absorption peak blue-shifted from 539.0 nm of the system of PBI-L with TEA to 537.6 nm of the system of PBI-L with TEA and HCl. Meanwhile, the relative absorption intensity ratio of the system of PBI-L with TEA and HCl around 537.6 nm was obviously larger than that of the system of PBI-L with TEA around 539.0 nm. These were caused by the transition of PBI-L from soluble states to aggregated states in the aqueous solution that contained TEA and HCl. In the aqueous solution of PBI-L after a heating, cooling, and aging process, the corresponding absorption peaks were found at about 499.3 and 534.2 nm, respectively. The relative absorption intensity ratio around 534.2 nm was also larger than that of the system of PBI-L with TEA. In addition, a new absorption peak around 469.6 nm was also found. These mean the formation of H-aggregated nanostructures by PBI-L in the aqueous solution after a heating, cooling, and aging process. The fluorescence excitation spectra (Figure 8b and Figure S30a) of PBI-L in the three systems for the emissions at 550.0 and 590.0 nm were very different from their UV-Vis spectra. The maximum excitation peak was found at about 531.3 nm, which means that the maximum fluorescence quantum yield would be obtained with an excitation around 531.3 nm. Meanwhile, the corresponding excitation peaks of the PBI-L shifted from 531.3 and 492.0 nm in the solution with TEA to 532.1 and 495.3 nm in the solution after a heating, cooling, and aging process, and to 532.4 and 495.1 nm in the solution with TEA and HCl, respectively. The corresponding excitation peaks of the PBI-L red-shifted from 464.1 nm in the solution with TEA to 466.9 nm in the solution after a heating, cooling, and aging process, but blue-shifted from 464.1 nm in the solution with TEA to 463.5 nm in the solution with TEA and HCl, respectively. The relative emission ratio of the PBI-L solution with TEA at 550.0 and 590.0 nm, excited by 492.0 nm, was smaller than the corresponding values of the PBI-L solution with TEA and HCl, and the PBI-L solution after a heating, cooling, and aging process. On the contrary, the relative emission ratio of the PBI-L solution with TEA at 550.0 and 590.0 nm, excited by the wavelength around 464.1 nm, was larger than the corresponding values of the PBI-L solution with TEA and HCl, and the PBI-L solution after a heating, cooling, and aging process. This means that the formation of nanostructures could enhance the S_1-0_ electronic vibration. The fluorescence emission spectra (Figure 8c) of PBI-L in the three systems excited by 430.0 nm were also very different. There was no emission peak around 478.4 nm in the PBI-L solution after a heating, cooling, and aging process, which may be due to the formation of H-aggregates. On the contrary, the corresponding emission peak of the PBI-L solution with TEA and HCl around 481.1 nm was found. This demonstrates that the arrangement of the perylene skeleton of the PBI-L system with TEA and HCl was different from that of the PBI-L system after a heating, cooling, and aging process. The corresponding emission peaks of the PBI-L solution with TEA around 550.6 and 589.9 nm blue-shifted to 546.8 and 588.1 nm in the PBI-L solution after a heating, cooling, and aging process, and red-shifted to 551.7 and 592.0 nm in the PBI-L solution with TEA and HCl, respectively. The relative emission ratio of the PBI-L solution with TEA around 589.9 nm was larger than the corresponding values of the PBI-L solution with TEA and HCl and the PBI-L solution after a heating, cooling, and aging process. The emission ratio of the PBI-L solution with TEA around 479.1 nm, excited by 450.0 nm (Figure S30b), was smaller than that excited by 430.0 nm. Meanwhile, the emission peak of the PBI-L solution with TEA and HCl around 481.1 nm disappeared.

The patterns of the UV-Vis spectra (Figure 8d) of the PBI-D solutions with TEA or TEA-HCl were similar to those of the PBI-L in the corresponding solutions. However, the relative absorption ratio around 469.1 nm in the PBI-D solution after a heating, cooling, and aging process further increased, while the absorption peak around 534.2 nm disappeared. This means that the PBI-D was more favorable to the formation of the H-aggregates. The patterns of the fluorescence excitation spectra (Figure 8e and Figure S30c) of the PBI-D in the solution with TEA and HCl and the solution after a heating, cooling, and aging process were similar to those of the PBI-L in the corresponding systems. The emission ratios of the PBI-D solution with TEA at 550.0 and 590.0 nm, excited by the wavelengths around 495.2 and 468.0 nm, are different from the corresponding values of the PBI-L solution with TEA. The positions of the corresponding emission peaks (Figure 8f and Figure S30d) of the three PBI-D solutions around 547.9 and 590.9 nm are subtly different from those of the three PBI-L solutions. The relative emission ratio of the PBI-D solution with TEA around 590.9 nm is smaller than the corresponding values of the self-assembly systems. The emission ratios of the PBI-D solutions around 481.8 nm, excited by 450.0 nm, are still smaller than the corresponding values of the PBI-D solutions around 485.3 nm excited by 430.0 nm.

The UV-Vis spectrum (Figure 8g) of the PBI-β solution with TEA is similar to that of the PBI-β solution with TEA and HCl. The absorption peak around 533.3 nm in the PBI-β solution without TEA and HCl is smaller than the absorption peaks around 540.2 nm in the other two PBI-β solutions. Meanwhile, the relative absorption ratio around 533.3 nm is much larger than the corresponding values for the other two PBI-β solutions. In the solutions without TEA and HCl, the relative absorption ratio of the PBI-β around 470.4 nm is much smaller than the corresponding values of the PBI-L and PBI-D. This is probably because the solubility of the PBI-β in deionized water was worse than the solubilities of the PBI-L and PBI-D in deionized water. The H-aggregation-type molecular packing of the PBI-β was not sufficient during the heating, cooling, and aging process. The patterns and regularities of the fluorescence excitation spectra (Figure 8h and Figure S30e) of the three PBI-β solutions between 400.0 and 580.0 nm for the emissions at 550.0 and 590.0 nm were similar to those of the corresponding PBI-L solutions. In the deionized water of PBI-β with TEA, there was a broad and strong fluorescence emission band between 470.0 and 720.0 nm excited by 430.0 nm (Figure 8i). The fluorescence emissions at about 480.6, 512.3, 545.8, and 591.2 nm were all very strong. In the deionized water of PBI-β with TEA and HCl, there were also four fluorescence emission peaks. However, the emission ratios at about 479.7 and 511.2 nm observably decreased, which is probably due to the formation of the nanostructures. In the deionized water of PBI-β without TEA and HCl, the emission peaks around 482.5 and 512.0 nm were also observed, which further indicates that the H-aggregation of the PBI-β was not sufficient. Meanwhile, the emission ratios of the PBI-β solution without TEA and HCl at about 482.5 and 512.0 nm were smaller than the corresponding values of the other two PBI-β solutions. This may result from the stronger H-aggregation of the PBI skeleton in the PBI-β solution without TEA and HCl. As the excitation wavelength is fixed at 450.0 nm, the patterns of the emission spectra (Figure S30f) of the three PBI-β systems are similar to their corresponding spectra excited by 430.0 nm. However, the emission ratios of the three PBI-β systems at about 480.6 and 512.3 nm excited by 450.0 nm are smaller than the corresponding values excited by 430.0 nm.

The above results show that the molecular packings of the PBI were highly dependent on the conformation of the small groups linked to the imide group, solvent polarity, and the self-assembly processes, which further affected the morphologies and optical properties of the nanostructures.

## 3. Conclusions

In summary, a series of 2D ribbon-like nanostructures and hierarchical nanostructures were fabricated by the self-assembly of the chiral alanine-decorated PBI in different solvents. The morphologies and optical properties of these nanostructures were demonstrated to be highly dependent on the solvent’s dissolving capacity, the chirality of alanine, and preparation methods. With the increase in the solvent’s dissolving capacity and the steric hindrance of the chiral alanine, the π-π stacking interaction of the PBI skeleton weakened, and the molecular packing of the PBI skeleton became more regular. The small chiral alanine moiety is unable to induce chirality in the nanostructures, owing to its low steric hindrance and the dominant strong π-π stacking interaction of the PBI skeleton. It was demonstrated that the hierarchical nanostructures are formed by the reorganization of the 2D ribbon-like nanostructures. The size of the 2D ribbon-like nanostructures and the amount of the hierarchical nanostructures increased with the decrease in the solvent’s dissolving capacity. In the alcoholic solutions, the UV-Vis absorption peaks and fluorescence emission peaks of the alanine-decorated PBI showed slight changes with variations in the solvent’s dissolving capacity and differences in the chirality of alanine. However, the corresponding absorption ratios and emission ratios changed notably. As the solvent’s dissolving capacity and the steric hindrance of the chiral alanine decreased, the emission ratios around 618.7 nm decreased, whereas those between 460.0 and 520.0 nm increased. A weaker π-π stacking interaction and better H-aggregated arrangement of the PBI skeleton could reduce the low-wavelength fluorescence intensity. In aqueous solutions, the UV-Vis absorption spectra and fluorescence emission spectra of the alanine-decorated PBI exhibited significant differences compared to those in alcoholic solutions and displayed a stronger dependence on the chirality of alanine. The heating, cooling, and aging process was conducive to the formation of H-aggregation of the PBI skeleton. The region of spectral overlap of the PBI solutions increases with the decrease in the dissolving capacity of the solvent and the steric hindrance of the chiral alanine. This study provides a perspective to tailor the morphologies of the nanostructures formed by the small π-conjugated molecules with poor solubility, and to comprehend the relationship between the optical properties of the nanostructures and the π-π stacking interaction, solvent, and chiral groups, which is useful for the design of sensors and photocatalysts.

## Data Availability

The data are contained within the article.

## Supplementary Materials

The following supporting information can be downloaded at: https://www.mdpi.com/article/10.3390/molecules29194610/s1, S1: Materials; S2: Synthesis and characterization of the chiral alanine-decorated perylene bisimides; S3: Preparation of the nanostructures; S4: Characterization; S5: The statistical sizes of the nanostructures; Figures S1–S3: The ^1^H NMR of the alanine-decorated PBI with TEA in D_2_O; Figures S4–S6: The MALDI-TOF MS of the alanine-decorated PBI; Figures S7–S9: The FT-IR spectra of the alanine-decorated PBI; Figure S10: The TEM images of the nanostructures formed by the PBI-L in *i*-PrOH (c = 0.0001 mg·mL^−1^) after a heating, cooling and aging process; Figure S11: The HRTEM and SAED images of the nanostructures formed by the PBI-L in *i*-PrOH (c = 0.0001 mg·mL^−1^) after a heating, cooling and aging process, (a) the 2D nanostructures, (b) the dumbbell-shaped nanostructures, (c,d) the SAED images of the dumbbell-shaped nanostructures. Figure S12: The statistical distributions of (a) the length, (b) the diameter of the ends and (c) the diameter of the central parts of the dumbbell-shaped nanostructures formed by the PBI-L in *i*-PrOH (c = 0.0001 mg·mL^−1^) after a heating, cooling and aging process; Figure S13: The AFM images of the 2D ribbon-like nanostructures formed by the PBI-L in i-PrOH (c = 0.0001 mg·mL^−1^) after a heating, cooling and aging process; Figure S14: Front, top and side views of molecular conformation and the corresponding sizes of the PBI-L, calculated with energy minimization (MM2) by ChemBio 3D Ultra, (a) the contour height of the perylene skeleton, (b) the contour width of the PBI-L (c) the contour height of the PBI-L; Figure S15: The fluorescence excitation spectra (at an emission band of 574 nm) (a,c,e) and the fluorescence emission spectra (excited by 450 nm) (b,d,f) of the PBI-L (a,b), PBI-D (c,d) and PBI-β (e,f) in alcoholic solvents (0.0001 mg·mL^−1^) after a heating, cooling and aging process; Figure S16: The TEM images of the nanostructures formed by the PBI-L in ethanol (a,b) and methanol (c,d) (c = 0.0001 mg·mL^−1^) after a heating, cooling and aging process; Figure S17: The statistical distributions of (a) the length of the straw-like nanostructures, (b) the diameter of the ends and (c) the diameter of the central parts of the rod-like nanostructures formed by the PBI-L in ethanol (c = 0.0001 mg·mL^−1^) after a heating, cooling and aging process; Figure S18: The TEM images of the nanostructures formed by the PBI-D in *i*-PrOH (a,b), ethanol (c,d) and methanol (e,f) (c = 0.0001 mg·mL^−1^) after a heating, cooling and aging process; Figure S19: The statistical distributions of the nanostructures formed by the PBI-D in three alcoholic solutions (c = 0.0001 mg·mL^−1^) after a heating, cooling and aging process, (a) the length and (b) width of the 2D ribbon-like nanostructures in *i*-PrOH, (c) the length and (d) width of the 2D ribbon-like nanostructures in ethanol, (e) the diameter of the straw-like nanostructures in methanol; Figure S20: The TEM images of the nanostructures formed by the PBI-β in *i*-PrOH (a,b), ethanol (c,d) and methanol (e,f) (c = 0.0001 mg·mL^−1^) after a heating, cooling and aging process. Figure S21: The statistical distributions of the nanostructures formed by the PBI-β in three alcoholic solutions (c = 0.0001 mg·mL^−1^) after a heating, cooling and aging process, (a) the length and (b) width of the 2D ribbon-like nanostructures in *i*-PrOH, (c) the length of the 2D nanostructures in ethanol, (d) the length of the spindle-like nanostructures in methanol; Figure S22: The probable molecular packing of the PBI in the 2D ribbon-like nanostructures formed by the PBI-β in *i*-PrOH and the PBI-D in *i*-PrOH and ethanol; Figure S23: The TEM images of the nanostructures formed by the (a,d,e) PBI-L, (b,e,g) PBI-D and (c,f,h) PBI-β in different conditions, (a–f) the aqueous solutions (c = 0.0001 mg·mL^−1^) underwent a heating, cooling and aging process, (e,g,h) the aqueous solutions (c = 1.0 mg·mL^−1^) underwent a ionization–neutralization process; Figure S24: The statistical distributions of the nanostructures formed by the PBI in aqueous solutions (c = 0.0001 mg·mL^−1^) after a heating, cooling and aging process, (a) the length and (b) width of the 2D ribbon-like nanostructures formed by PBI-L, (c) the width of the 2D nanostructures formed by PBI-β; Figure S25: The AFM images of the 2D ribbon-like nanostructures formed by the PBI in the aqueous solutions (c = 0.1 mg·mL^−1^) underwent a ionization–neutralization process, (a,d) PBI-L, (b,e) PBI-D, (c,f) PBI-β; Figure S26: The statistical distributions of the nanostructures formed by the PBI in aqueous solutions (c = 0.1 mg·mL^−1^) underwent a ionization–neutralization process, (a) the length and (b) width of the 2D ribbon-like nanostructures formed by PBI-L, (c) the length and (d) width of the 2D ribbon-like nanostructures formed by PBI-D, (e) the length and (f) width of the 2D ribbon-like nanostructures formed by PBI-β; Figure S27: The FT-IR spectra of the 2D ribbon-like nanostructures formed by the PBI in the aqueous solutions (c = 0.1 mg·mL^−1^) underwent an ionization–neutralization process, (a) PBI-L, (b) PBI-D, (c) PBI-β. Figure S28: The XRD spectra of the 2D ribbon-like nanostructures formed by the PBI in the aqueous solutions (c = 0.1 mg·mL^−1^) underwent a ionization–neutralization process. Figure S29: The probable unit cell of the 2D ribbon-like nanostructures formed by the PBI in the aqueous solutions (c = 0.1 mg·mL^−1^) underwent a ionization–neutralization process. Figure S30: The fluorescence excitation spectra (at an emission band of 590 nm) (a,c,e) and the fluorescence emission spectra (excited by 450 nm) (b,d,f) of the PBI-L (a,b), PBI-D (c,d) and PBI-β (e,f) in aqueous solutions through different processes; Table S1: Summary Data of the nanostructures; Tables S2–S4: Summary of data of the UV-Vis spectra, the fluorescence excitation spectra and the fluorescence emission spectra of the PBI in different solvents. Table S5: Summary of the XRD data of the nanostructures formed through an ionization–neutralization method.
