# Supplementary material for: Morphology Control and Spectral Study of the 2D and Hierarchical Nanostructures Self-Assembled by the Chiral Alanine-Decorated Perylene Bisimides
Source: Molecules. 2024 Sep 28;29(19):4610. doi: 10.3390/molecules29194610 (PMC11477776; doi:10.3390/molecules29194610)
Supplement: Supplementary file 1 [file molecules-29-04610-s001.zip › molecules-3199550-supplementary.pdf]

Morphology control and spectral study of the 2D and hierarchical nanostructures self-assembled by the chiral alanine-decorated perylene bisimides

Rui Qi[1], Xiaotian Huang[1], Ting Yang[1], Peng Luo[1], Wensheng Qi[1], Yin Zhang[1], Haimei Yuan[1], Hongmei Li[1], Jian Wang[1], Baohua Liu[1] and Songzhi Xie[1,*]

[1]College of Food and Biological Engineering, Chengdu University, Chengdu 610106, P. R. China.

[*]Correspondence: xiesongzhi@cdu.edu.cn (S. Xie)

Supporting Information

Contents

1. Materials
2. Synthesis and characterization of the chiral alanine-decorated perylene bisimides
3. Preparation of the nanostructures
4. Characterization
5. The statistical sizes of the nanostructures
6. Self-assembly of the PBI-L in *i*-PrOH
7. Effect of the solvent on the self-assembly of the PBI-L
8. Effect of the chirality on the self-assembly of the PBI
9. Self-assembly of the chiral PBI in aqueous solutions

1. Materials

3,4,9,10-Perylene tetracarboxylic dianhydride (PTCDA), L-alanine, D-alanine, β-alanine, imidazole (AR), Dimethyl Formamide (DMF, AR), triethylamine (TEA, AR), HCl (36%, AR), methanol (HPLC), ethanol (HPLC) and isopropanol (*i*-PrOH, HPLC) were purchased from Aladdin and used without further purification. Dilute hydrochloric acid was obtained by adding the concentrated hydrochloric acid (36 wt%, AR) to the deionized water.

2. Synthesis and characterization of the chiral alanine-decorated perylene bisimides

The three chiral alanine-decorated perylene bisimides (PBI-L, PBI-D and PBI-β) were synthesized as shown in Scheme 1. PTCDA (0.784 g, 2.00 mmol), alanine acid (0.356 g, 4.00 mmol) and imidazole (2.800 g) were added into a flame-dried 100 mL one-neck round-bottom flask. After being flooded with argon gas for 30 minutes, the mixture was heated to 120 °C for 12 h, and then cooled to 90 °C. 3 ml of the deionized water was added to the system. After being heated at 90 °C for 1 h, the system was cooled down to 25 °C. The above synthesis processes were carried out in an argon atmosphere with a balloon. The pH value of the reaction mixture was then adjusted to 2 with HCl (10 wt%). The black-brown precipitate was collected by suction filtration and then washed with the deionized water until the pH value of the filtrate became 7.0. Then, the collected precipitate was dissolved in deionized water with an adequate amount of triethylamine (TEA). After suction filtration, excessive HCl was added to the filtrate that contained TEA. The newly formed precipitate was collected once again by suction filtration and subsequently washed with deionized water until the pH value of the filtrate reached 7.0. Finally, the pure precipitate was dried in high vacuum at 60 °C for 12 h.

For PBI-L (1.010 g, yield= 88.6%), ^{1}H NMR (**Figure S1**, 400 MHz, D_2O/TEA, δ): 7.80-7.43 (d, 8H), 5.31 (m, 2H) and 1.67 (d, 6H). MALDI-TOF MS (**Figure S4**): m/z = 536 (M^+, calcd). FT-IR (cm^{-1}) (**Figure S7**): ν =1694.8 and 1640.8 (-C=O, amide groups).

For PBI-D (1.040 g, yield= 91.2%), ^{1}H NMR (**Figure S2**, 400 MHz, D_2O/TEA, δ): 7.73-7.31 (m, 8H), 5.30 (m, 2H) and 1.65 (d, 6H). MALDI-TOF MS (**Figure S5**): m/z = 536 (M^+, calcd). FT-IR (cm^{-1}) (**Figure S8**): ν =1696.1 and 1653.7 (-C=O, amide groups).

For PBI-β (0.984 g, yield= 86.3%), ^{1}H NMR (**Figure S3**, 400 MHz, D_2O/TEA, δ): 7.53 (m, 8H), 4.05 (m, 4H) and 2.47 (m, 4H). MALDI-TOF MS (**Figure S6**): m/z = 536 (M^+, calcd). FT-IR (cm^{-1}) (**Figure S9**): ν =1689.0 and 1653.0 (-C=O, amide groups).

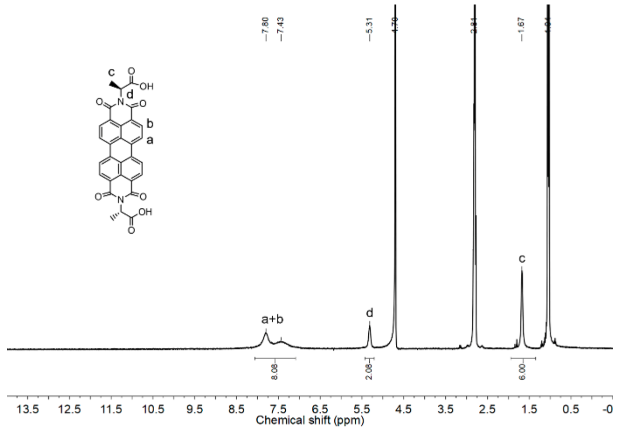

Figure S1. The ^1H NMR of the L-alanine-decorated PBI with TEA in D_2O

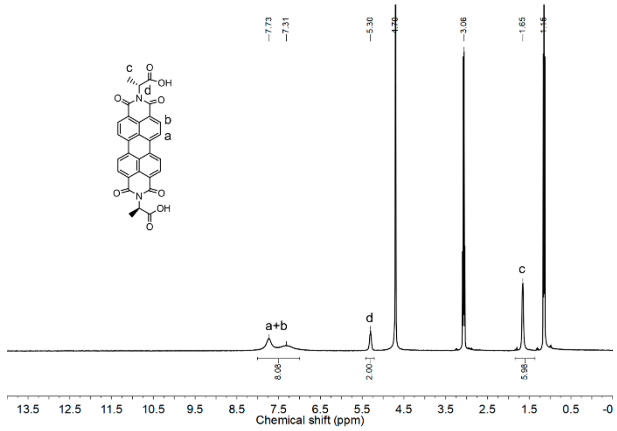

Figure S2. The ^1H NMR of the D-alanine-decorated PBI with TEA in D_2O

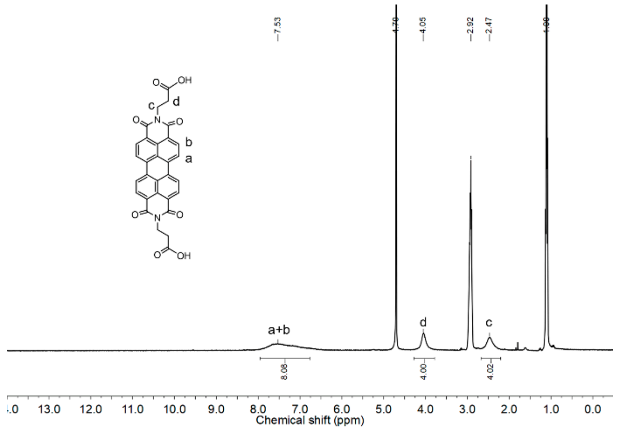

Figure S3. The ^1H NMR of the β-alanine-decorated PBI with TEA in D_2O

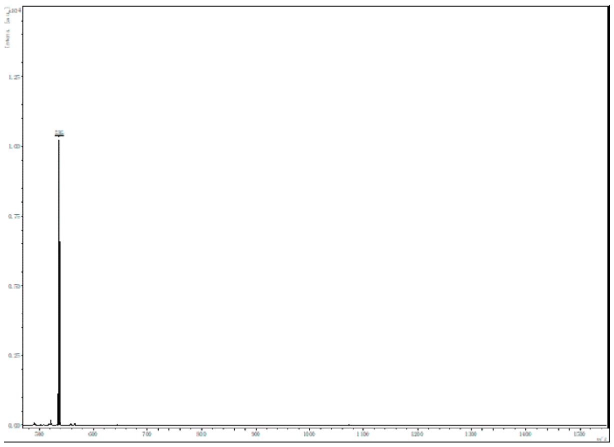

Figure S4. The MALDI-TOF MS of the PBI-L, the molecular weight of the PBI-L is 536 M/z.

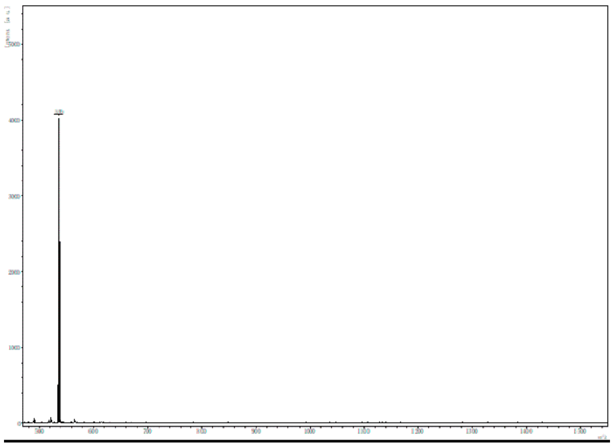

Figure S5. The MALDI-TOF MS of the PBI-D, the molecular weight of the PBI-D is 536 M/z.

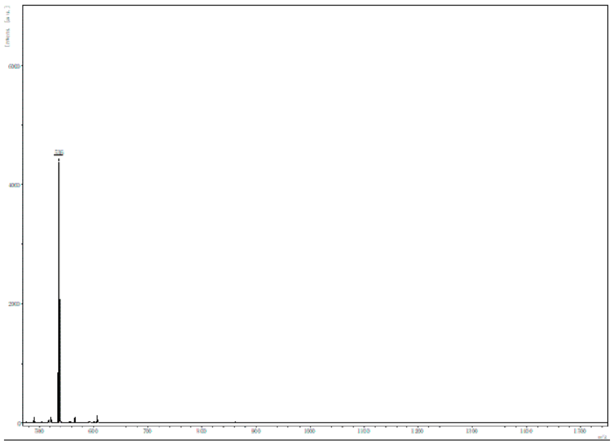

Figure S6. The MALDI-TOF MS of the PBI-β, the molecular weight of the PBI-β is 536 M/z.

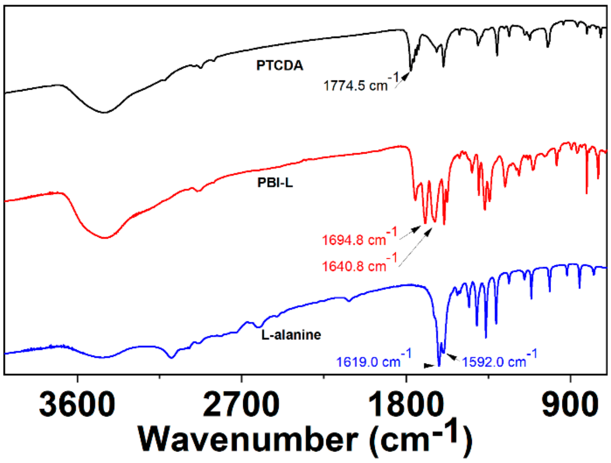

Figure S7. The FT-IR spectra of the PTCDA, PBI-L, and L-alanine.

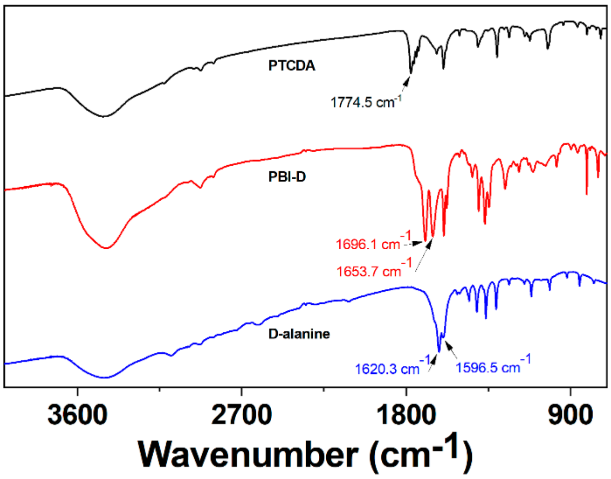

Figure S8. The FT-IR spectra of the PTCDA, PBI-D and D-alanine.

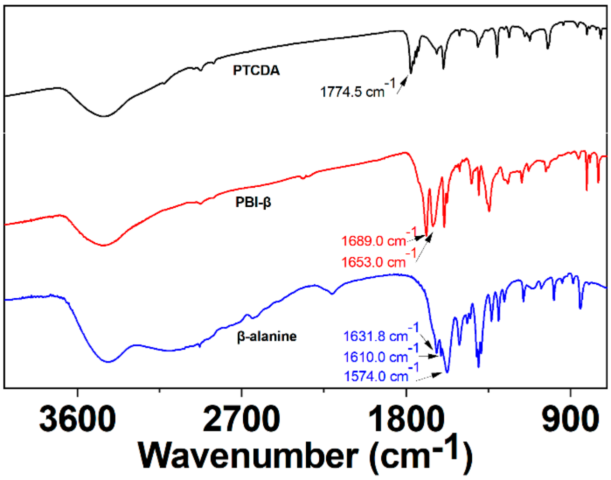

Figure S9. The FT-IR spectra of the PTCDA, PBI-β and β-alanine.

3. Preparation of the nanostructures

Method A. 0.1 mg of the PBI and 1000 ml of alcoholic solvents or deionized water were added into a 2000 ml of bottles with a lid. The sealed bottles were heated to a certain temperature (60

°C for methanol, 75 °C for ethanol, 80 °C for *i*-PrOH) for 2 h in water bath, and then slowly cooled down to room temperature, followed by aging for 12 h at 25 °C.

Method B. 1.0 mg of the PBI was dissolved in 6.0 ml of deionized water containing 10 μl of TEA in a 20 ml vial. Following 30 minutes of ultrasonic agitation, 220 μl of 1M HCl was slowly added to the vial while stirring. Subsequently, the solutions were dialyzed for 3 days. 10 ml of the final assembly solutions were prepared by adding deionized water to the dialyzed solutions.

4. Characterization

^{1}H nuclear magnetic resonance (^{1}H NMR). The ^{1}H NMR spectra were acquired using a Bruker AVANCE 400 MHz spectrometer, employing D_2O containing a specific quantity of TEA as the solvent.

Fourier transform infrared spectrometer (FT-IR). Fourier transform infrared spectra (FT-IR) were obtained on a Perkin-Elmer system 2000 FT-IR spectrometer.

Matrix-assisted laser desorption ionization time-of-flight mass spectrometry (MALDI-TOF MS). MALDI-TOF MS spectra were recorded on a Bruker Flex Series MALDI-TOF MS in positive-ion reflectron mode with an accelerating voltage of 20 kV. The samples were prepared by mixing a DMF solution of 2′, 6′-dihydroxyacetophenone matrix solution (0.25 M) and a DMF solution of PBI (1 mg·mL^{-1}).

Transmission Electron Microscopy (TEM). 10 μl of the polymer solutions were quickly obtained from the well-shaken solutions at the room temperature using a 100 microliter pipette, and then were drop-casted onto the carbon-coated copper grids as soon as possible. The samples were prepared after evaporating the solvents at the room temperature. TEM images were obtained on a Hitachi HT7800 microscope operating at 100 kV and equipped with an AMF-5016 charge-coupled device camera.

High-resolution transmission electron microscope (HRTEM) and Selected area electron diffraction (SAED) were obtained on the JEM-F200. The samples were prepared as same as the TEM samples.

Atomic Force Microscopy (AFM). The AFM samples were prepared by drop casting 10 μl of the well-shaken polymer solutions onto the mica sheets, and then evaporating solvents. Imaging

was performed on a Bruker Dimension Icon in ScanAsyst in Air, using ScanAsyst -Air as probe. Images were obtained by nanoscope analysis.

X-ray powder diffraction. The X-ray powder diffraction measurements were carried out on a Bruker AXS D8 ADVANCE X-ray diffractometer equipped with a nickel-filtered Cu-Kα radiation (λ = 1.5418 Å). The samples for X-ray diffraction measurement were prepared as follows. 1000 ml of the aqueous solutions with PBI (0.1 mg·mL^{-1}) were first filtrated, and then the obtained precipitates were dried in vacuum at 60 ºC for 12 h. The dried precipitates were tested by the X-ray diffraction measurement.

UV-Vis Absorption Spectra (UV-Vis). The UV-Vis absorption spectra were recorded by a SHIMADZU UV3600 spectrophotometer. 3 ml of the well-shaken solutions during a heating, cooling and aging process were directly transformed into the sealed quartz glass cuvette and tested at room temperature. Specially, the UV-Vis absorption spectra of the aqueous solutions with the PBI concentration at 0.1 mg·mL^{-1} during an ionization and neutralization process were obtained as follows. 30 μl of the well-shaken solutions were diluted by adding 30 ml of the deionized water in the 50 ml of vials. After being shaken up, 3 ml of the new obtained solutions were quickly transformed into the sealed quartz glass cuvette and tested at room temperature.

Fluorescence spectroscopy. The fluorescence spectra were carried out using a Perkin-Elmer FL8500 fluorescence spectrometer with an air filter. The excitation slit and emission slit widths were 5 nm, the scanning speed was 1200 nm min^{-1}. The phosphorescence lifetimes were obtained after an exposure time of 100 ms. 3 ml of the well-shaken solutions during a heating, cooling and aging process were directly transformed into the sealed quartz glass cuvette and tested at room temperature. Specially, the UV-Vis absorption spectra of the aqueous solutions with the PBI concentration at 0.1 mg·mL-1 during an ionization and neutralization process were obtained as follows. 30 μl of the well-shaken solutions were diluted by adding 30 ml of the deionized water in the 50 ml of vials. After being shaken up, 3 ml of the new obtained solutions were quickly transformed into the sealed quartz glass cuvette and tested at room temperature.

5. The statistical sizes of the nanostructures

The sizes of each sample were obtained by analyzing two hundred micelles from TEM images using Digital Micrograph software (US Gatan company). The length and width were used to characterize the scale of the 2D nanostructures. The length and diameter were used to

characterize the scale of the dumbbell-shaped and rod-like nanostructures. The number-average length (L_n), the weight-average length (L_w), the number-average width (W_n), the weight-average width (W_w) of the 2D nanostructures, the L_n, L_w, the number-average diameter (D_n), the weight-average diameter (D_w) of the dumbbell-shaped and rod-like nanostructures were calculated by the following equations: (where L_i, W_i and D_i are the sizes of individual micelle, respectively, N_i are the number of L_i, W_i and D_i).

$$\boldsymbol{L}_n = \frac{\sum_{i=1}^{N} N_i L_i}{\sum_{i=1}^{N} N_i}$$

$$\boldsymbol{L}_w = \frac{\sum_{i=1}^{N} N_i {L_i}^2}{\sum_{i=1}^{N} N_i L_i}$$

$$\boldsymbol{W}_n = \frac{\sum_{i=1}^{N} N_i W_i}{\sum_{i=1}^{N} N_i}$$

$$\boldsymbol{W}_w = \frac{\sum_{i=1}^{N} N_i {W_i}^2}{\sum_{i=1}^{N} N_i W_i}$$

$$\boldsymbol{D}_n = \frac{\sum_{i=1}^{N} N_i D_i}{\sum_{i=1}^{N} N_i}$$

$$\boldsymbol{D}_w = \frac{\sum_{i=1}^{N} N_i {D_i}^2}{\sum_{i=1}^{N} N_i D_i}$$

6. Self-assembly of the PBI-L in *i*-PrOH

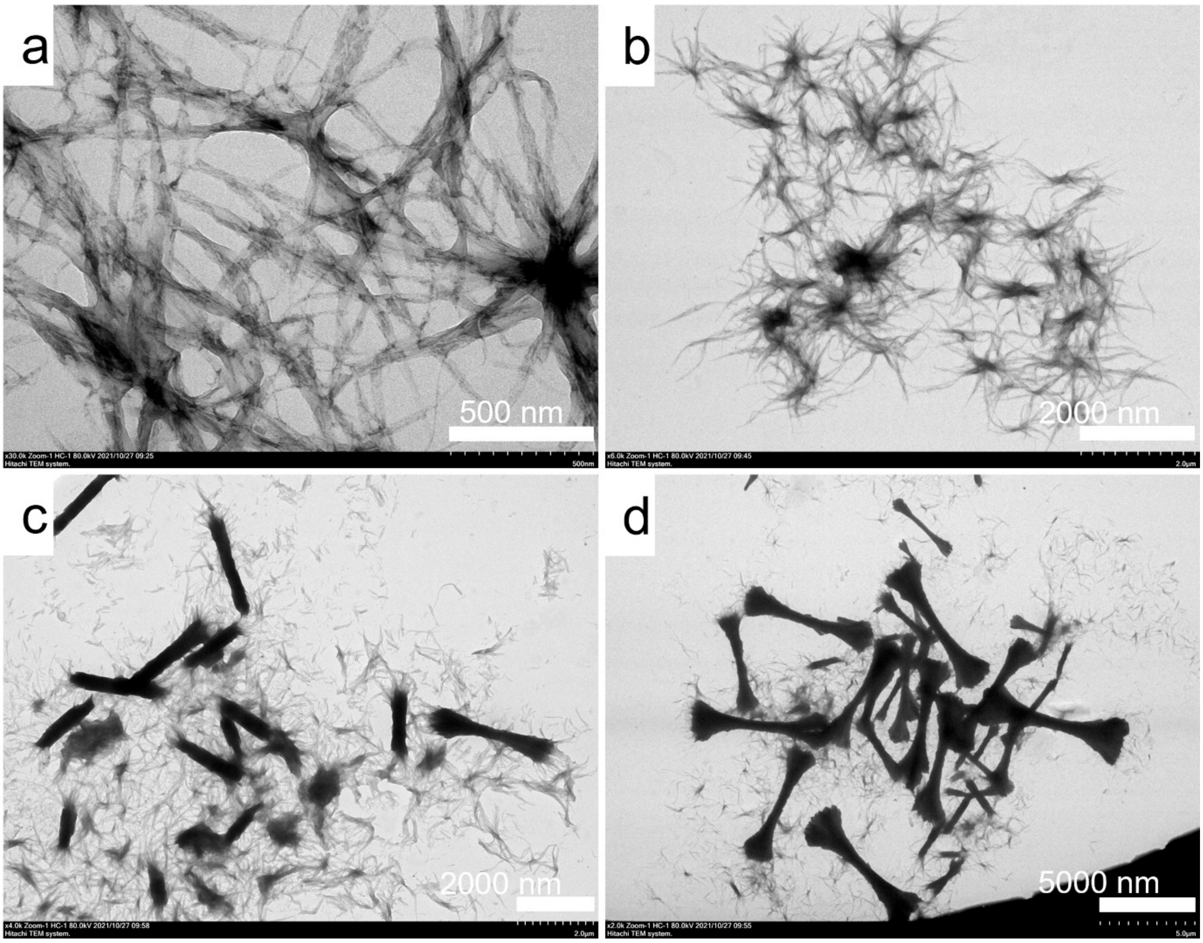

Figure S10. The TEM images of the nanostructures formed by the PBI-L in *i*-PrOH (c= 0.0001 mg·mL^{-1}) after a heating, cooling and aging process.

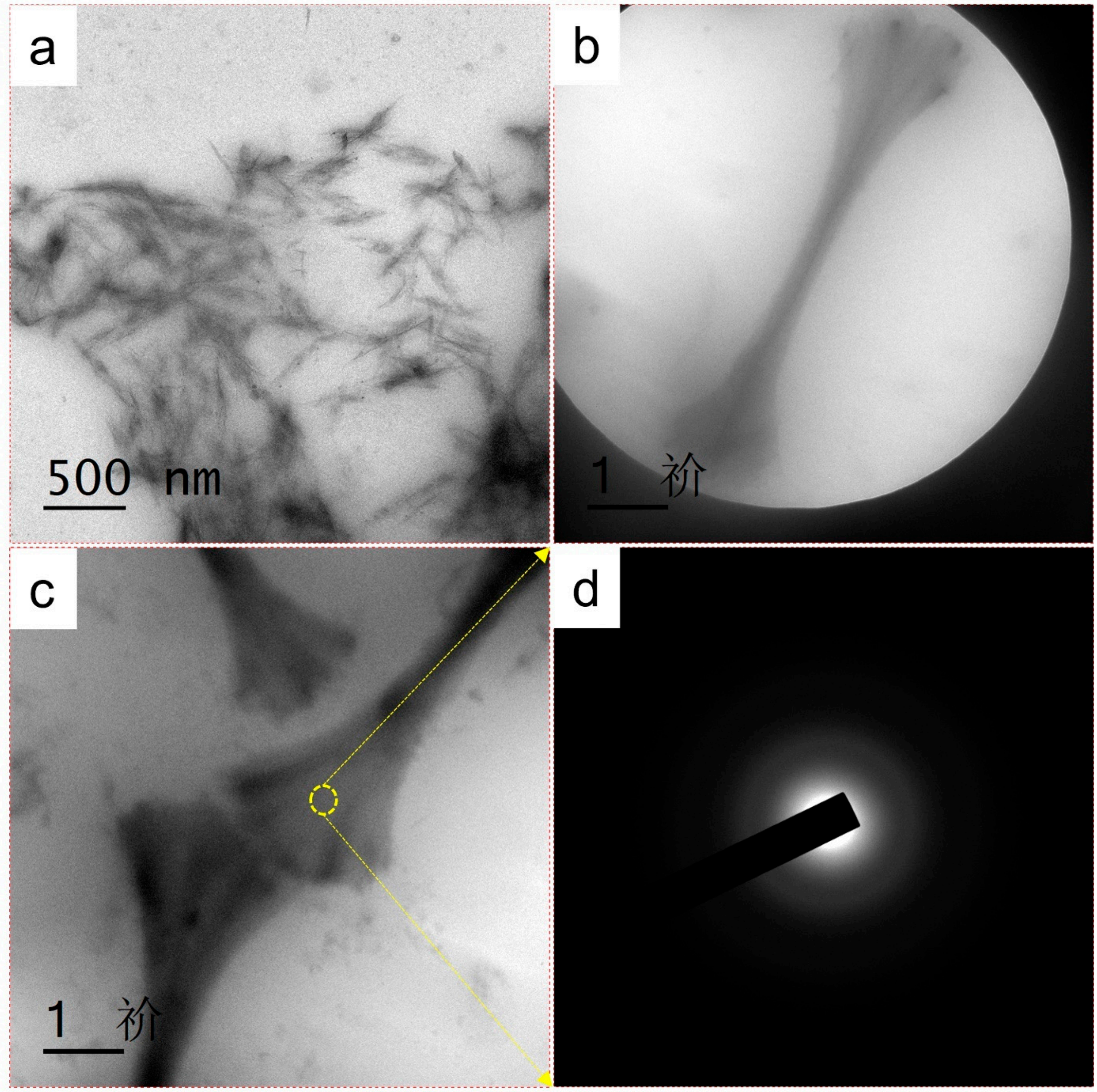

Figure S11. The HRTEM and SAED images of the nanostructures formed by the PBI-L in *i*-PrOH (c= 0.0001 mg·mL-1) after a heating, cooling and aging process, a) the 2D nanostructures, b) the dumbbell-shaped nanostructures, c, d) the SAED images of the dumbbell-shaped nanostructures.

Table S1. Summary Data of the nanostructures.

PBI	Solvents	T (°C)	Nanostructures	L_n [a)] (nm)	L_w [b)]/L_n	W_n [c)] (nm)	W_w [d)]/W_n	$D_{1,n}$ [e)] (nm)	$D_{1,w}$ [f)]/$D_{1,n}$	$D_{2,n}$ (nm)	$D_{2,w}$/$D_{2,n}$
**PBI-L**	Deionized water	80	2D nanostructures	890	1.20	76	1.09				
**PBI-L**	TEA-HCl	25	2D nanostructures	120	1.19	11	1.05				
**PBI-L**	CH_3CH_2OH	75	Straw-like nanostructures	1258	1.07						
			Rod-lke nanostructures	3745	1.08					268	1.24
**PBI-L**	*i*-PrOH	80	dumbbell-shaped nanostructures	3039	1.33			1071	1.33	358	1.07
**PBI-D**	TEA-HCl	25	2D nanostructures	183	1.19	9	1.05				
**PBI-D**	CH_3OH	60	Straw-like nanostructures					2100	1.13		
**PBI-D**	CH_3CH_2OH	75	2D nanostructures	725	1.13	35	1.10				
**PBI-D**	*i*-PrOH	80	2D nanostructures	253	1.17	27	1.08				
**PBI-β**	Deionized water	80	2D nanostructures			49	1.15				
**PBI-β**	TEA-HCl	25	2D nanostructures	221	1.26	19	1.06				
**PBI-β**	CH_3OH	60	spindle-like nanostructures	1009	1.07						
**PBI-β**	CH_3CH_2OH	75	2D nanostructures	368	1.36						
**PBI-β**	*i*-PrOH	80	2D nanostructures	146	1.10	21	1.09				

[a] L_n is the number-average length of the 2D nanostructures and hierarchical nanostructures; [b] L_w is the weight-average length of the 2D nanostructures; [c] W_n is the number-average width of the 2D nanostructures; [d] W_w is the weight-average width of the 2D nanostructures; [e] D_n is the number-average diameter of the hierarchical nanostructures; [f] D_w the weight-average length of the hierarchical nanostructures.

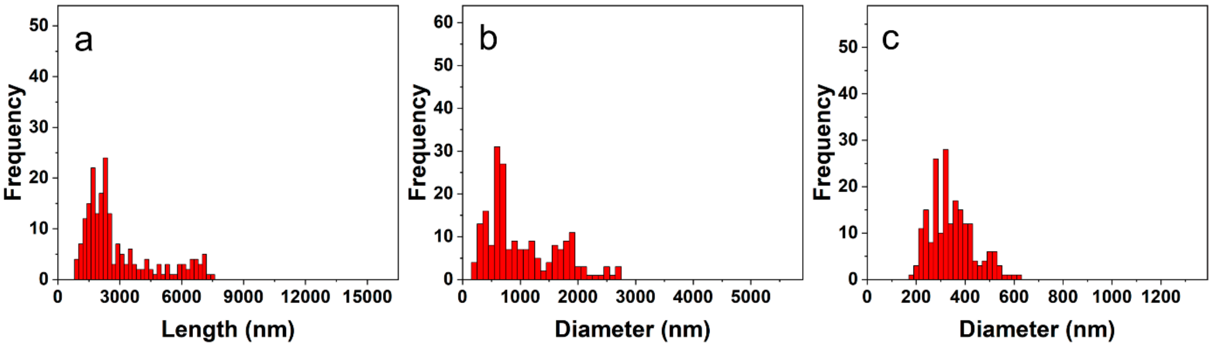

Figure S12. The statistical distributions of (a) the length, (b) the diameter of the ends and (c) the diameter of the central parts of the dumbbell-shaped nanostructures formed by the PBI-L in *i*-PrOH (c= 0.0001 mg·mL^{-1}) after a heating, cooling and aging process.

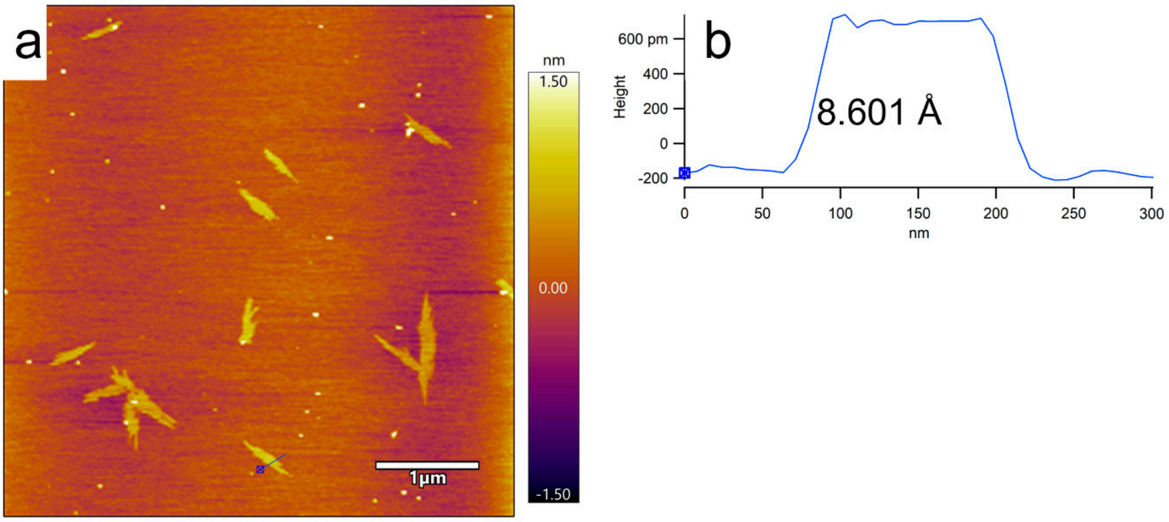

Figure S13. The AFM images of the 2D ribbon-like nanostructures formed by the PBI-L in *i*-PrOH (c= 0.0001 mg·mL^{-1}) after a heating, cooling and aging process.

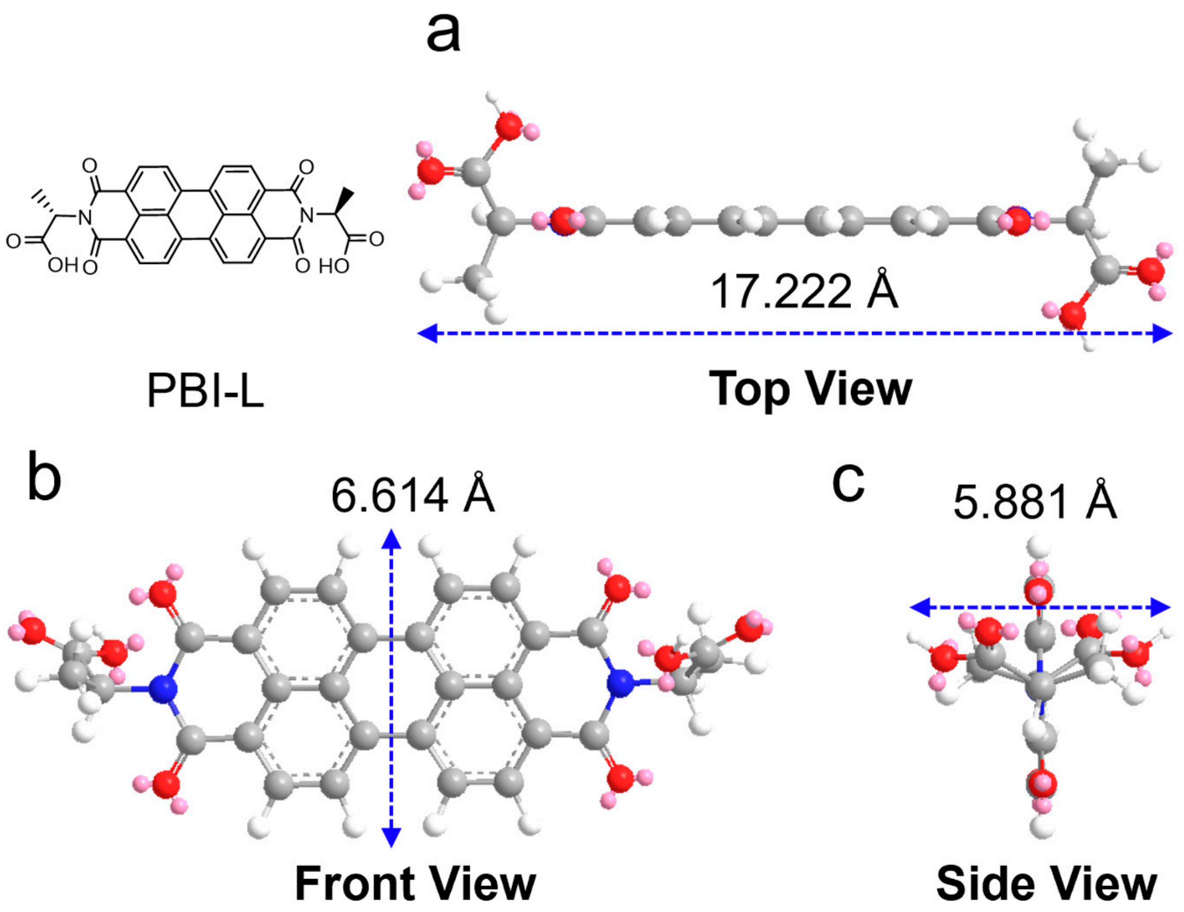

Figure S14. Front, top and side views of molecular conformation and the corresponding sizes of the PBI-L, calculated with energy minimization (MM2) by ChemBio 3D Ultra, (a) the contour height of the perylene skeleton, (b) the contour width of the PBI-L (c) the contour height of the PBI-L.

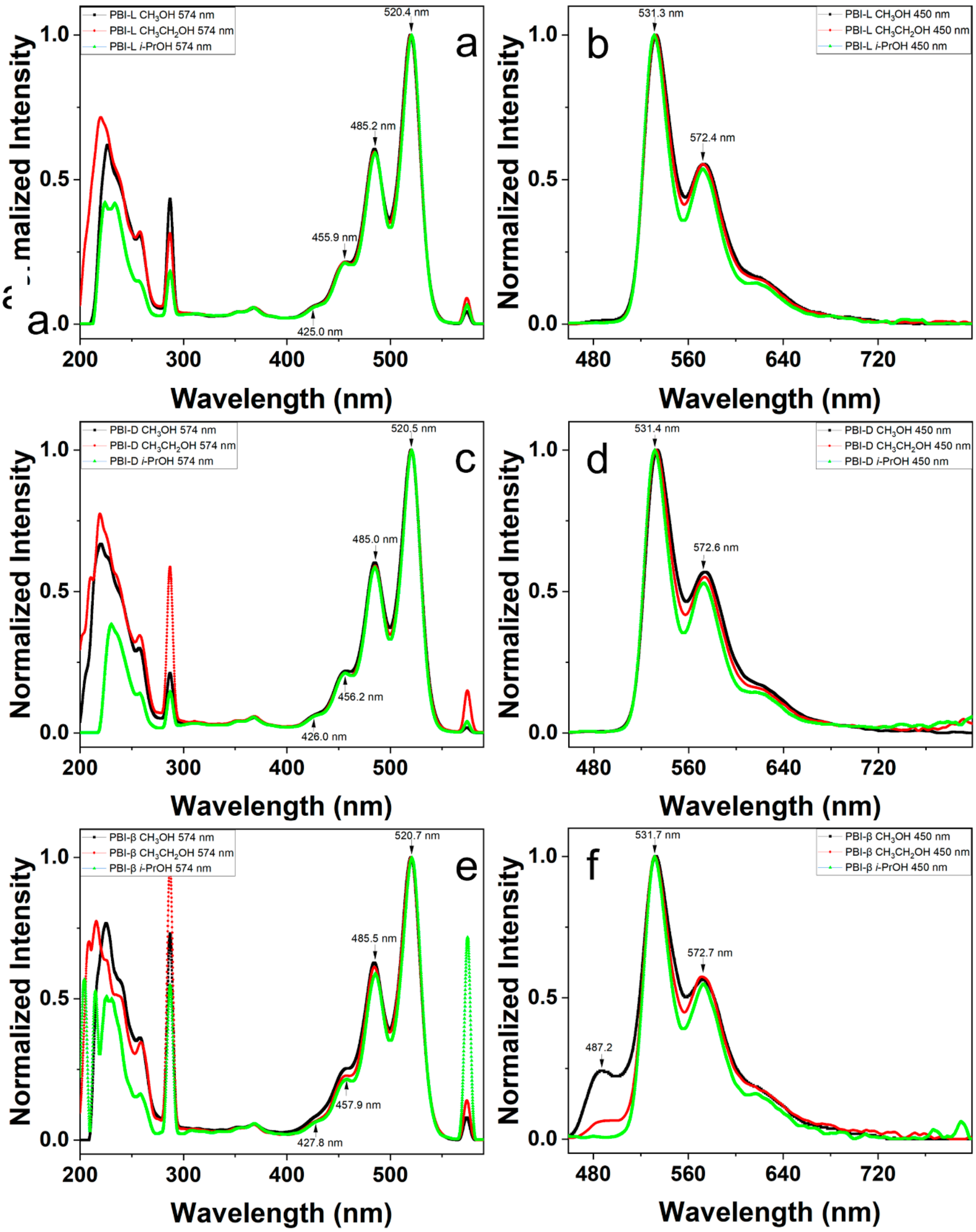

Figure S15. The fluorescence excitation spectra (at an emission band of 574 nm) (a, c, e) and the fluorescence emission spectra (excited by 450 nm) (b, d, f) of the PBI-L (a, b,), PBI-D (c, d) and PBI-β (e, f) in alcoholic solvents (0.0001 mg·mL^{-1}) after a heating, cooling and aging process.

7. Effect of the solvent on the self-assembly of the PBI-L

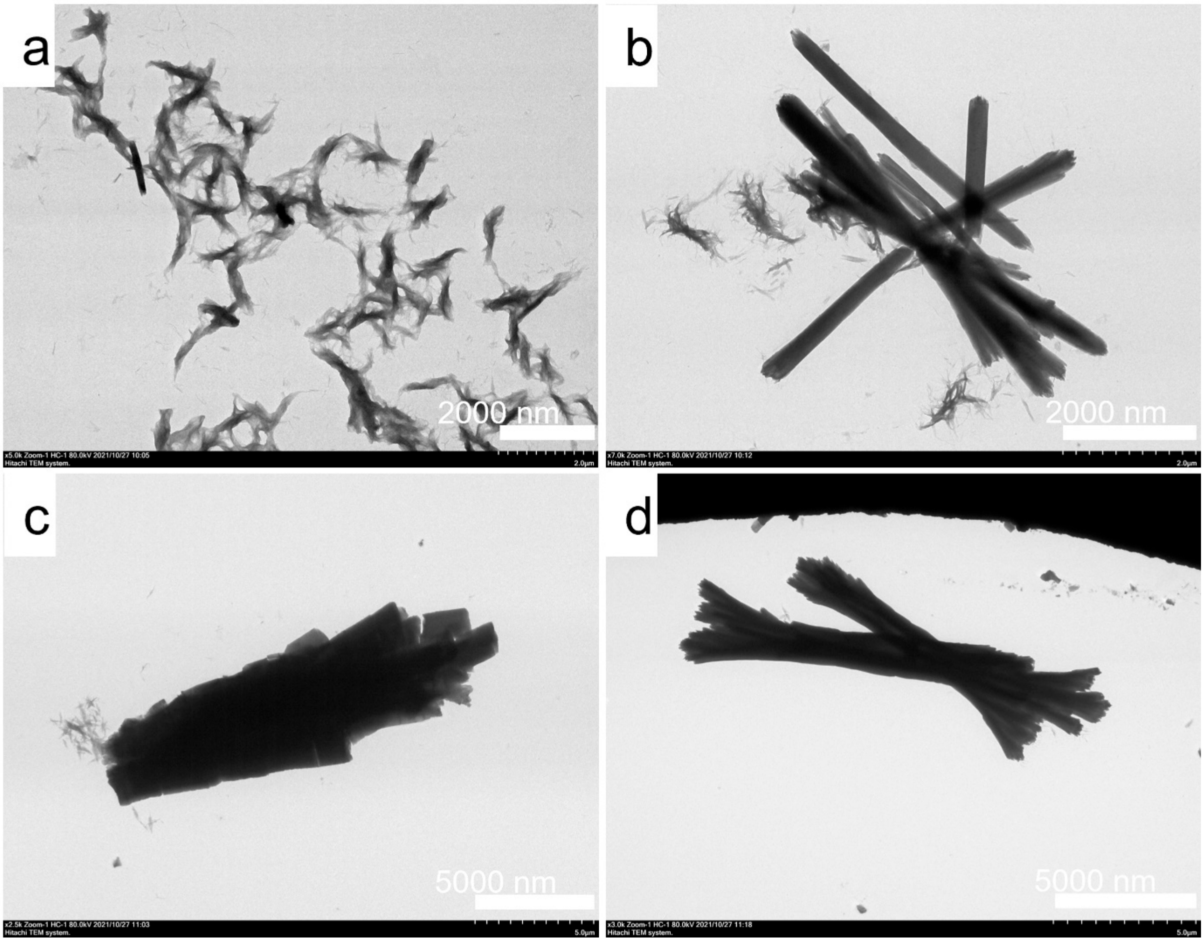

Figure S16. The TEM images of the nanostructures formed by the PBI-L in ethanol (a, b) and methanol (c, d) (c= 0.0001 mg·mL^{-1}) after a heating, cooling and aging process.

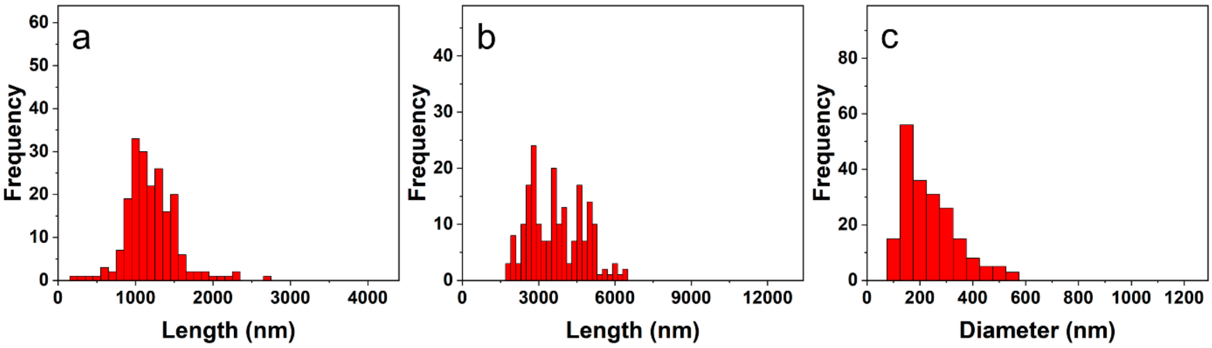

Figure S17. The statistical distributions of (a) the length of the straw-like nanostructures, (b) the diameter of the ends and (c) the diameter of the central parts of the rod-like nanostructures formed by the PBI-L in ethanol (c= 0.0001 mg·mL^{-1}) after a heating, cooling and aging process.

Table S2. Summary of data of the UV-Vis spectra of the PBI in different solvents

PBI	Solvents	$\lambda_{max,3}$ (nm)[a]	$A_{max,3}/A_{max}$[b]	$\lambda_{max,2}$ (nm)[a]	$A_{max,2}/A_{max}$[b]	$\lambda_{max,1}$ (nm)[a]	$A_{max,1}/A_{max}$[b]
**PBI-L**	Deionized water	469.6	0.748	499.3	1	534.2	0.728
**PBI-L**	TEA			501.0	1	539.0	0.709
**PBI-L**	TEA-HCl			501.4	1	537.6	0.768
**PBI-L**	CH_3OH	455.0	0.292	483.9	0.664	519.3	1
**PBI-L**	CH_3CH_2OH	456.0	0.492	485.0	0.812	520.0	1
**PBI-L**	*i*-PrOH	457.0	0.491	485.0	0.791	521.0	1
**PBI-D**	Deionized water	469.1	0.982	497.7	1		
**PBI-D**	TEA			501.0	1	537.0	0.744
**PBI-D**	TEA-HCl			500.6	1	536.3	0.805
**PBI-D**	CH_3OH	457.7	0.314	484.6	0.705	519.2	1
**PBI-D**	CH_3CH_2OH	459.0	0.646	485.0	0.880	520.0	1
**PBI-D**	*i*-PrOH	456.0	0.428	485.0	0.755	521.0	1
**PBI-β**	Deionized water	470.4	0.511	498.8	1	533.3	0.957
**PBI-β**	TEA			499.0	1	540.0	0.537
**PBI-β**	TEA-HCl			499.4	1	540.2	0.544
**PBI-β**	CH_3OH			483.5	0.731	519.4	1
**PBI-β**	CH_3CH_2OH	460.3	0.354	484.6	0.723	520.1	1
**PBI-β**	*i*-PrOH	459.0	0.335	486.0	0.729	521.0	1

[a]$\lambda_{max,1}$, $\lambda_{max,2}$ and $\lambda_{max,3}$ are the corresponding wavelengths of the UV-Vis absorption peaks, [b]$A_{max,1}$, $A_{max,2}$ and $A_{max,3}$ are the absorbance at $\lambda_{max,1}$, $\lambda_{max,2}$ and $\lambda_{max,3}$, respectively, A_{max} is the maximum absorbance.

Table S3. Summary of data of the fluorescence excitation spectra of the PBI in different solvents

PBI	Solvents	λ_{em} (nm)[a]	$\lambda_{ex,3}$ (nm)[b]	$F_{ex,3}/F_{ex,max}$[c]	$\lambda_{ex,2}$ (nm)[b]	$F_{ex,2}/F_{ex,max}$[c]	$\lambda_{ex,1}$ (nm)[b]	$F_{ex,1}/F_{ex,max}$[c]
**PBI-L**	Deionized water	550	466.9	0.228	495.3	0.593	532.1	1
		590	470.1	0.228	495.3	0.599	532.3	1
**PBI-L**	TEA	550	464.1	0.308	492.0	0.512	531.3	1
		590	464.8	0.315	492.0	0.524	531.4	1
**PBI-L**	TEA-HCl	550	463.5	0.293	495.1	0.614	532.4	1
		590	464.1	0.291	495.0	0.604	532.1	1
**PBI-L**	CH_3OH	534	456.3	0.215	484.5	0.598	519.6	1
		574	457.2	0.215	484.6	0.605	519.5	1
**PBI-L**	CH_3CH_2OH	534	456.5	0.216	484.7	0.599	520.1	1
		574	456.2	0.214	484.7	0.596	520.0	1
**PBI-L**	*i*-PrOH	534	456.6	0.211	485.2	0.560	520.5	1
		574	455.9	0.211	485.2	0.593	520.4	1
**PBI-D**	Deionized water	550	467.6	0.223	495.5	0.595	532.3	1
		590	468.7	0.225	495.3	0.595	532.5	1
**PBI-D**	TEA	550	468.0	0.239	495.2	0.600	532.2	1
		590	467.6	0.242	495.1	0.603	532.0	1
**PBI-D**	TEA-HCl	550	463.6	0.292	494.8	0.614	532.6	1
		590	463.7	0.299	494.8	0.626	531.6	1
**PBI-D**	CH_3OH	534	457.3	0.219	485.0	0.605	520.1	1
		574	457.5	0.220	485.0	0.603	520.1	1
**PBI-D**	CH_3CH_2OH	534	456.7	0.211	485.0	0.590	520.4	1
		574	456.5	0.211	485.2	0.590	520.3	1
**PBI-D**	*i*-PrOH	534	456.7	0.210	485.1	0.588	520.7	1
		574	456.2	0.211	485.0	0.587	520.5	1
**PBI-β**	Deionized water	550	467.4	0.246	495.3	0.601	532.1	1
		590	467.5	0.252	495.2	0.613	532.2	1
**PBI-β**	TEA	550	465.6	0.305	494.0	0.511	531.4	1
		590	467.4	0.275	493.9	0.522	531.6	1
**PBI-β**	TEA-HCl	550	463.5	0.321	494.7	0.611	531.9	1
		590	463.5	0.311	494.3	0.610	531.7	1
**PBI-β**	CH_3OH	534			484.3	0.613	519.8	1
		574			484.4	0.628	519.9	1
**PBI-β**	CH_3CH_2OH	534	456.6	0.221	484.8	0.599	520.1	1
		574	457.5	0.228	484.6	0.612	520.2	1
**PBI-β**	*i*-PrOH	534	457.1	0.214	485.3	0.596	520.8	1
		574	457.9	0.214	485.5	0.589	520.7	1

[a]λ_{em} is the fluorescence emission wavelength, [b]$\lambda_{ex,1}$, $\lambda_{ex,2}$ and $\lambda_{ex,3}$ are the corresponding excitation wavelengths of the emission peaks at λ_{em}, respectively, [c]$F_{ex,1}$, $F_{ex,2}$ and $F_{ex,3}$ are the fluorescence intensity of λ_{em} excited at $\lambda_{ex,1}$, $\lambda_{ex,2}$ and $\lambda_{ex,3}$, respectively, $F_{ex,max}$ is the maximum fluorescence intensity of λ_{em}.

Table S4. Summary of data of the fluorescence emission spectra of the PBI in different solvents

PBI	Solvents	λ_{ex} (nm)[a]	$\lambda_{em,1}$ (nm)[b]	$F_{em,1}/F_{em,max}$[c]	$\lambda_{em,2}$ (nm)[b]	$F_{em,2}/F_{em,max}$[c]	$\lambda_{em,3}$ (nm)[b]	$F_{em,3}/F_{em,max}$[c]	$\lambda_{em,4}$ (nm)[b]	$F_{em,4}/F_{em,max}$[c]
**PBI-L**	Deionized water	430			501.4	0.040	546.8	1	588.1	0.527
		450					547.3	1	588.5	0.551
**PBI-L**	TEA	430	478.4	0.151			550.6	1	589.9	0.865
		450	479.1	0.063			549.4	1	590.0	0.814
**PBI-L**	TEA-HCl	430	481.1	0.196			551.7	1	592.0	0.769
		450					546.6	1	589.7	0.542
**PBI-L**	CH_3OH	430	491.5	0.112			532.7	1	572.2	0.575
		450					532.9	1	573.3	0.554
**PBI-L**	CH_3CH_2OH	430					532.2	1	573.3	0.550
		450					532.3	1	572.4	0.552
**PBI-L**	*i*-PrOH	430					532.3	1	572.3	0.559
		450					531.3	1	572.4	0.536
**PBI-D**	Deionized water	430					549.3	1	590.9	0.860
		450					549.0	1	591.0	0.842
**PBI-D**	TEA	430					547.9	1	590.9	0.574
		450					548.1	1	590.0	0.642
**PBI-D**	TEA-HCl	430	485.3	0.108			551.5	1	591.2	0.948
		450	481.8	0.023			549.0	1	589.3	0.715
**PBI-D**	CH_3OH	430					533.5	1	573.3	0.594
		450					533.5	1	573.8	0.569
**PBI-D**	CH_3CH_2OH	430	491.3	0.034			532.6	1	574.9	0.551
		450					532.5	1	573.5	0.551
**PBI-D**	*i*-PrOH	430					532.6	1	572.4	0.576
		450					531.4	1	572.6	0.529
**PBI-β**	Deionized water	430	482.5	0.292			546.7	1	590.4	0.560
		450	482.8	0.137			547.6	1	590.2	0.574
**PBI-β**	TEA	430	480.6	0.826	512.3	0.737	545.8	1	591.2	0.524
		450	480.9	0.462	511.3	0.412	546.7	1	586.7	0.553
**PBI-β**	TEA-HCl	430	479.7	0.631	511.2	0.479	548.3	1	587.8	0.514
		450	479.7	0.327	516.2	0.244	546.4	1	586.5	0.541
**PBI-β**	CH_3OH	430	488.6	0.524			531.8	1	568.6	0.531
		450	487.2	0.242			531.7	1	572.7	0.567
**PBI-β**	CH_3CH_2OH	430	491.7	0.175			531.7	1	572.9	0.577
		450	496.9	0.066			531.8	1	571.3	0.574
**PBI-β**	*i*-PrOH	430	491.4	0.080			531.9	1	572.6	0.553
		450					531.7	1	572.7	0.549

[a]λ_{ex} is the fluorescence excitation wavelength, [b]$\lambda_{em,1}$, $\lambda_{em,2}$ and $\lambda_{em,3}$ are the corresponding wavelengths of the emission peaks excited at λ_{ex}, respectively, [c]$F_{em,1}$, $F_{em,2}$ and $F_{em,3}$ are the fluorescence intensity at $\lambda_{em,1}$, $\lambda_{em,2}$ and $\lambda_{em,3}$, respectively, $F_{ex,\,max}$ is the maximum fluorescence intensity.

8. Effect of the chirality on the self-assembly of the PBI

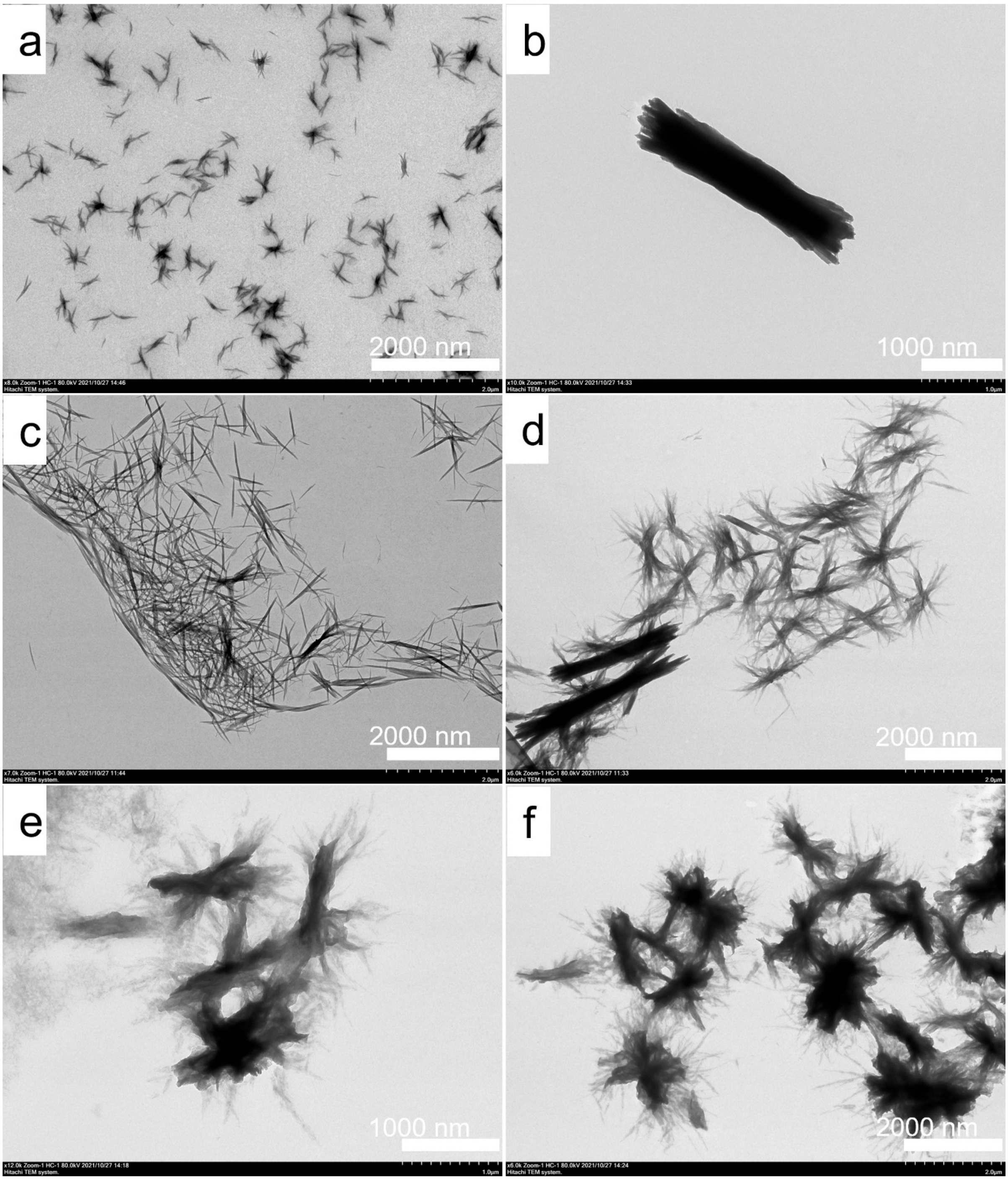

Figure S18. The TEM images of the nanostructures formed by the PBI-D in *i*-PrOH (a, b), ethanol (c, d) and methanol (e, f) (c= 0.0001 mg·mL^{-1}) after a heating, cooling and aging process.

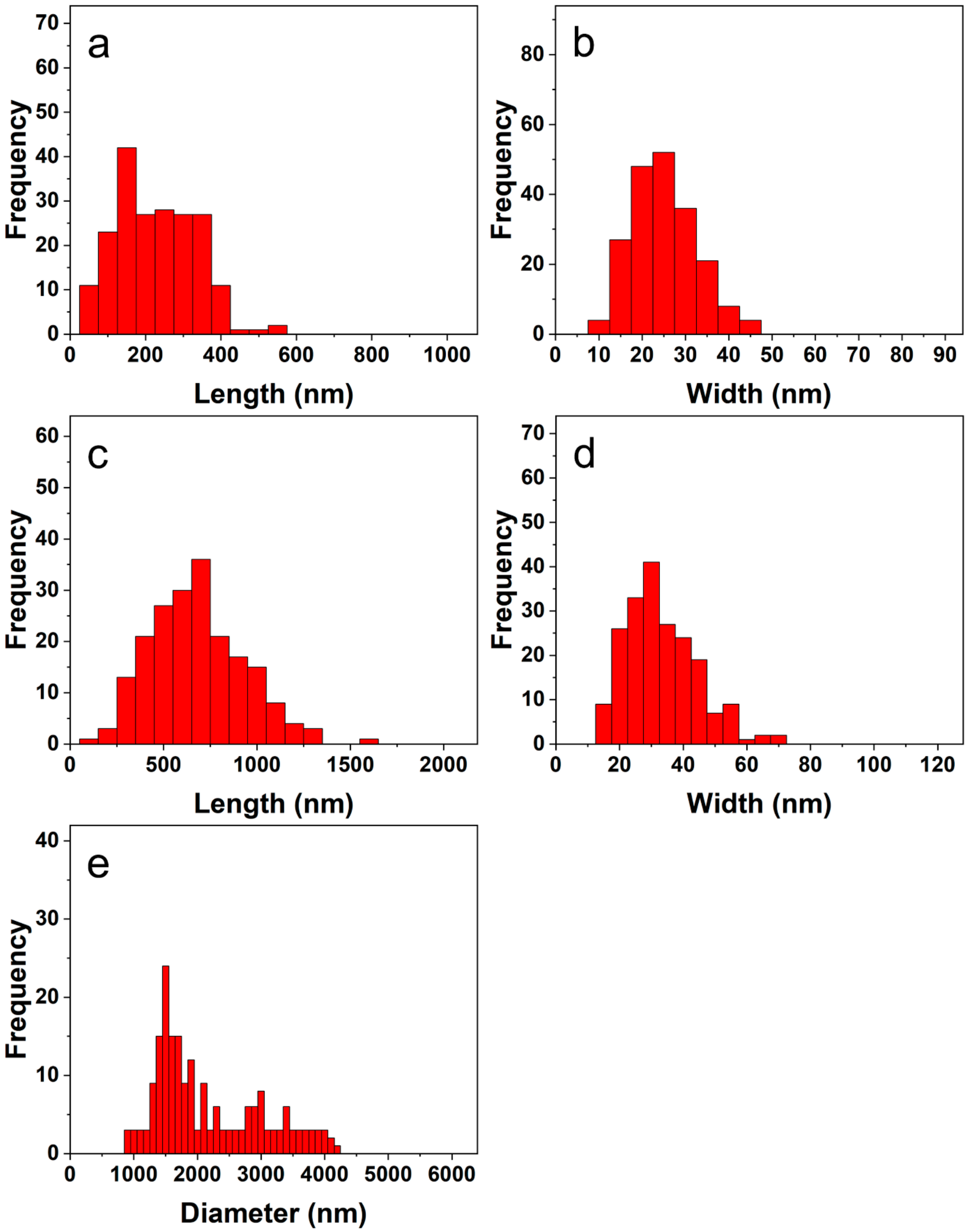

Figure S19. The statistical distributions of the nanostructures formed by the PBI-D in three alcoholic solutions (c= 0.0001 mg·mL^{-1}) after a heating, cooling and aging process, (a) the length and (b) width of the 2D ribbon-like nanostructures in *i*-PrOH, (c) the length and (d) width of the 2D ribbon-like nanostructures in ethanol, (e) the diameter of the straw-like nanostructures in methanol.

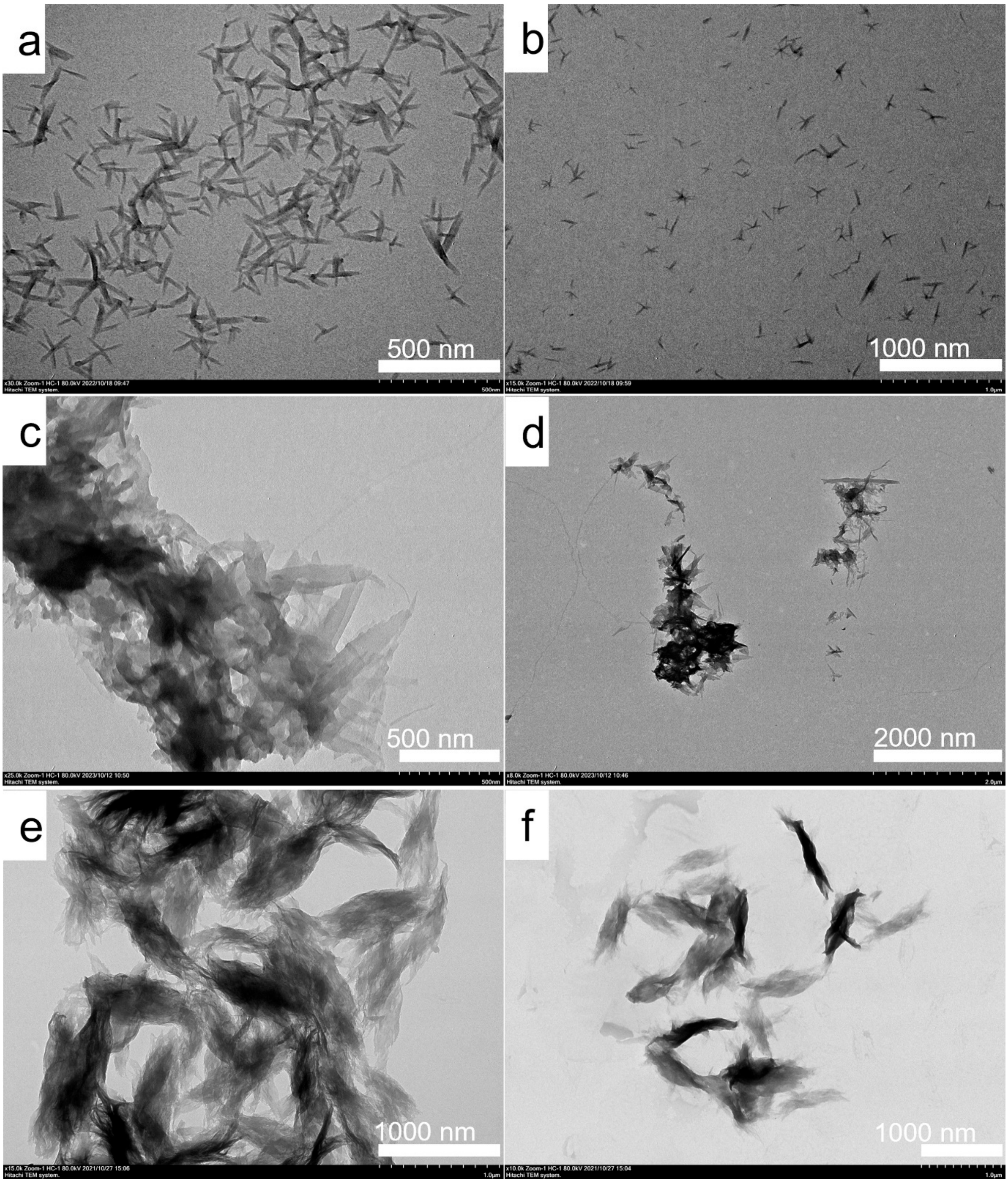

Figure S20. The TEM images of the nanostructures formed by the PBI-β in *i*-PrOH (a, b), ethanol (c, d) and methanol (e, f) (c= 0.0001 mg·mL^{-1}) after a heating, cooling and aging process.

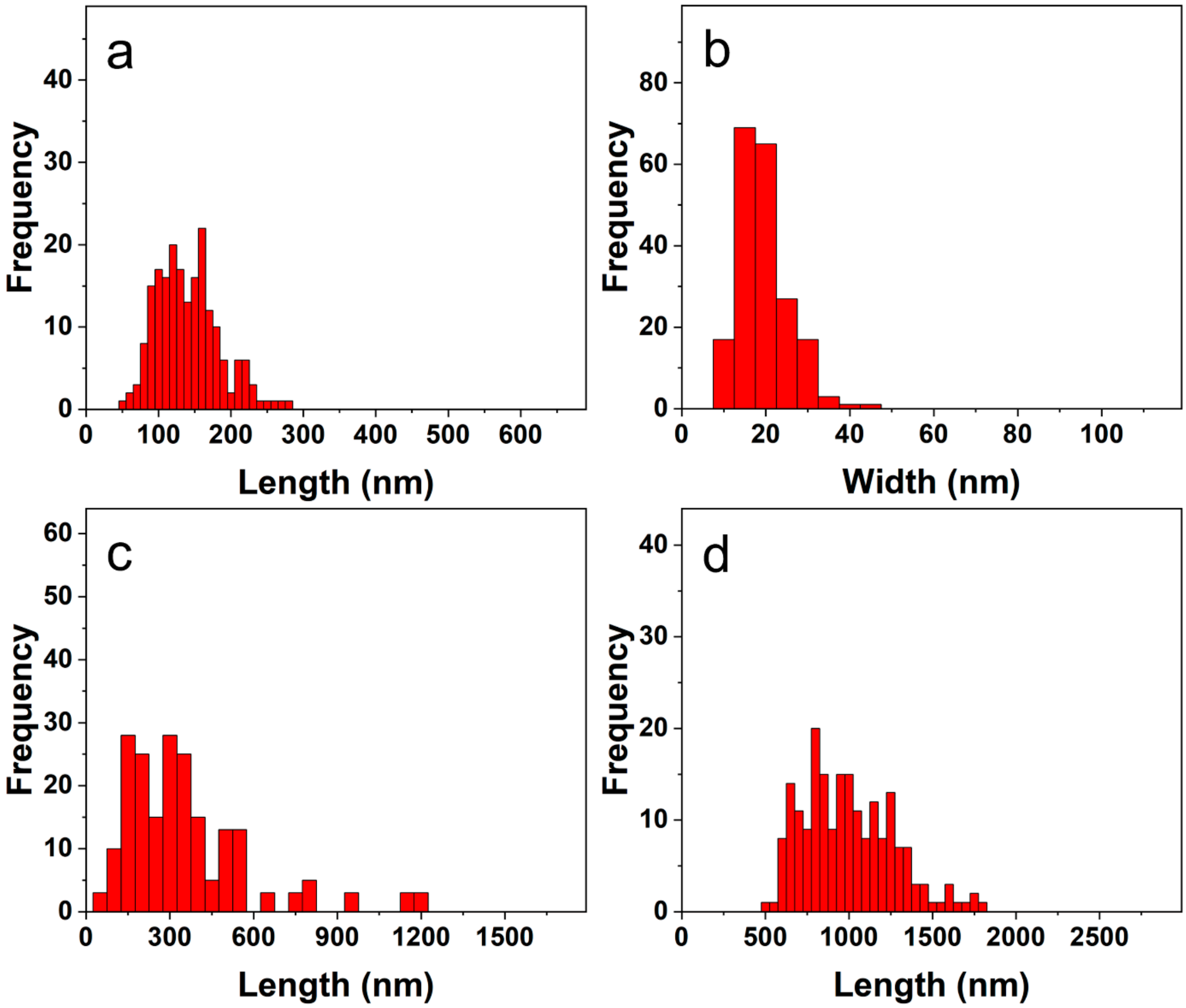

Figure S21. The statistical distributions of the nanostructures formed by the PBI-β in three alcoholic solutions (c= 0.0001 mg·mL^{-1}) after a heating, cooling and aging process, (a) the length and (b) width of the 2D ribbon-like nanostructures in *i*-PrOH, (c) the length of the 2D nanostructures in ethanol, (d) the length of the spindle-like nanostructures in methanol.

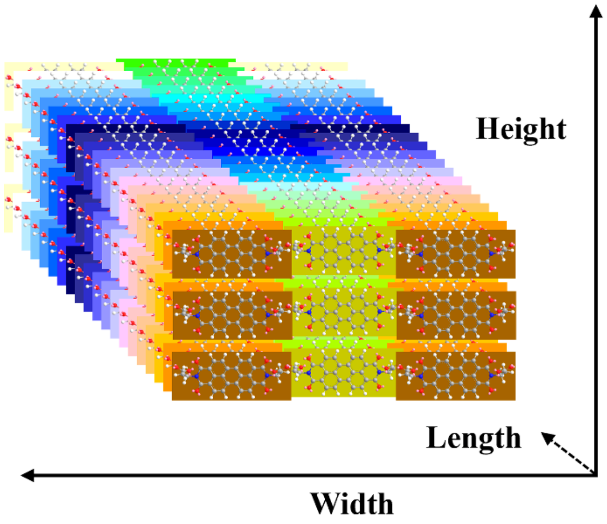

Figure S22. The probable molecular packing of the PBI in the 2D ribbon-like nanostructures formed by the PBI-β in *i*-PrOH and the PBI-D in *i*-PrOH and ethanol.

9. Self-assembly of the chiral PBI in aqueous solutions

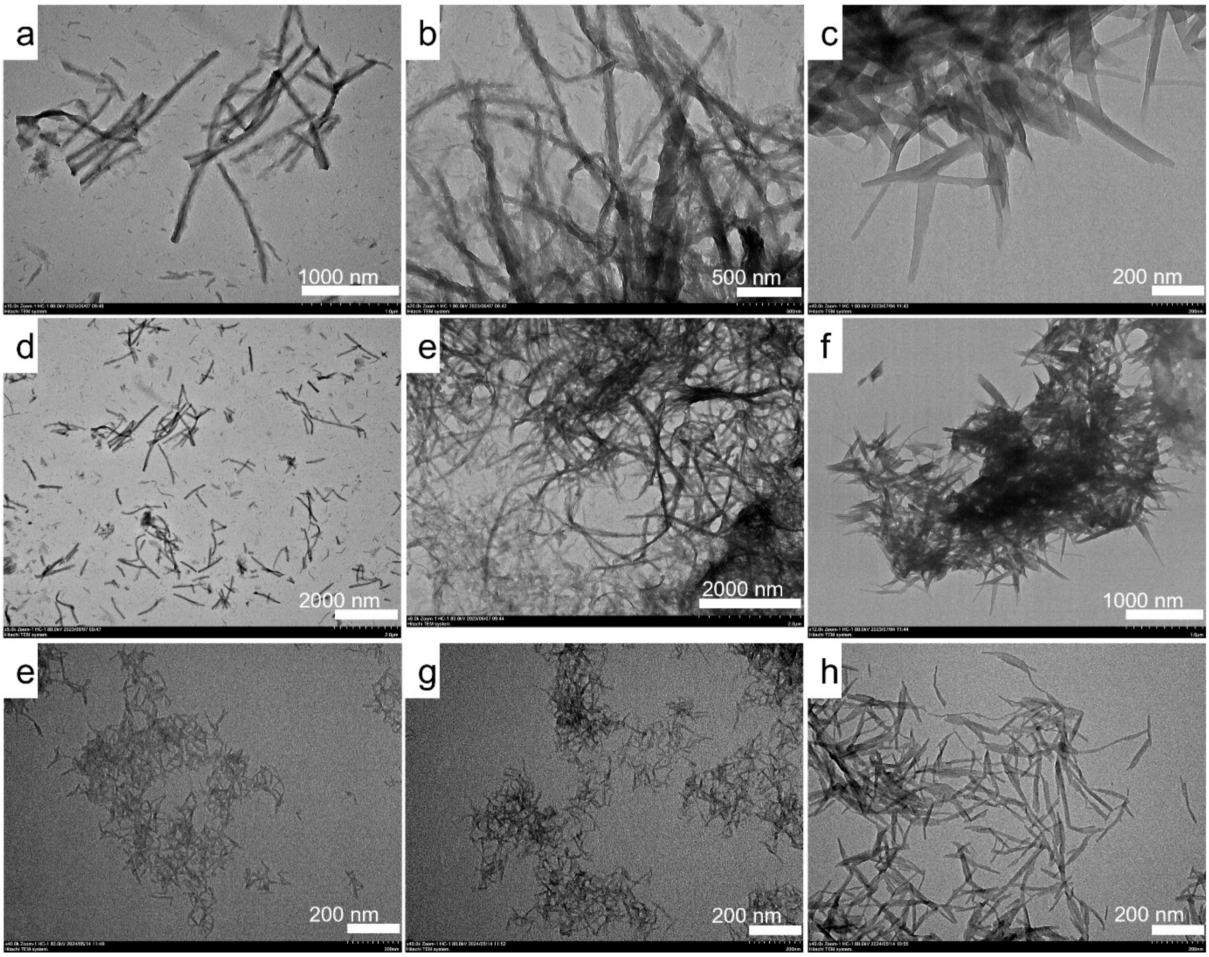

Figure S23. The TEM images of the nanostructures formed by the (a, d, e) PBI-L, (b, e, g) PBI-D and (c, f, h) PBI-β in different conditions, (a, b, c, d, e, f) the aqueous solutions (c= 0.0001

mg·mL^{-1}) underwent a heating, cooling and aging process, (e, g, h) the aqueous solutions (c= 0.1 mg·mL^{-1}) underwent a ionization-neutralization process.

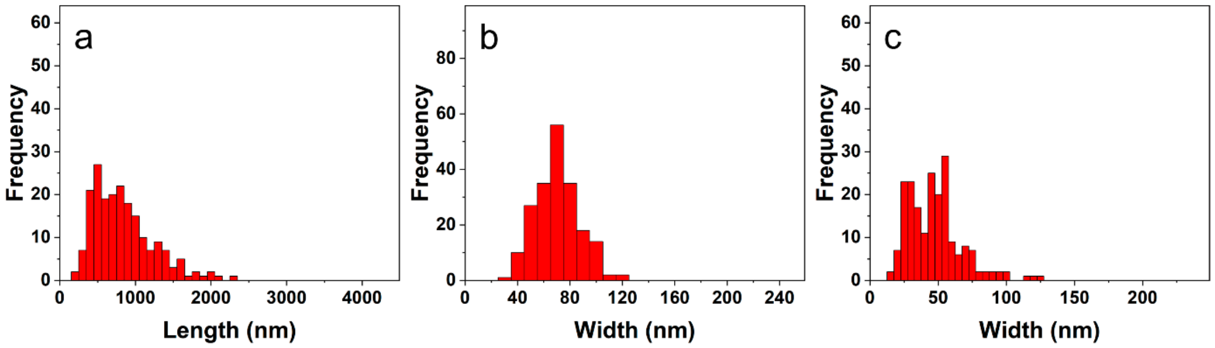

Figure S24. The statistical distributions of the nanostructures formed by the PBI in aqueous solutions (c= 0.0001 mg·mL^{-1}) after a heating, cooling and aging process, (a) the length and (b) width of the 2D ribbon-like nanostructures formed by PBI-L, (c) the width of the 2D nanostructures formed by PBI-β.

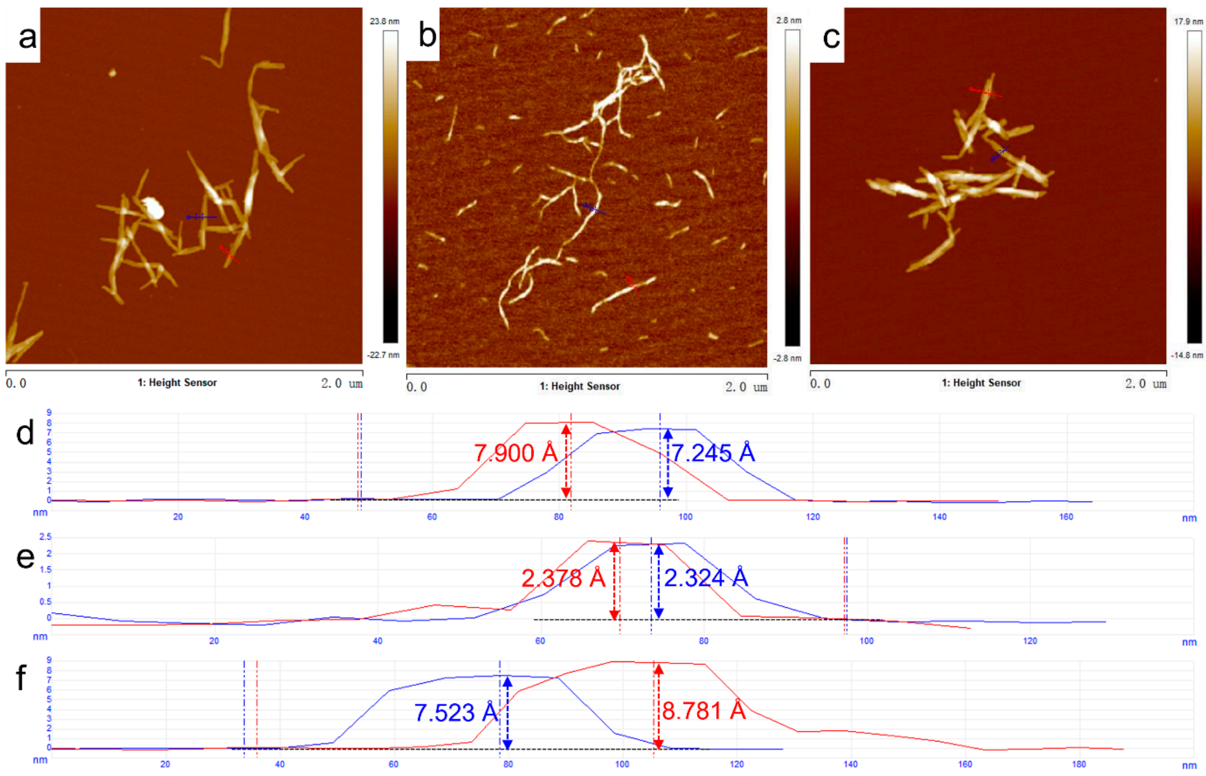

Figure S25. The AFM images of the 2D ribbon-like nanostructures formed by the PBI in the aqueous solutions (c= 0.1 mg·mL^{-1}) underwent a ionization-neutralization process, (a, d) PBI-L, (b, e) PBI-D, (c, f) PBI-β.

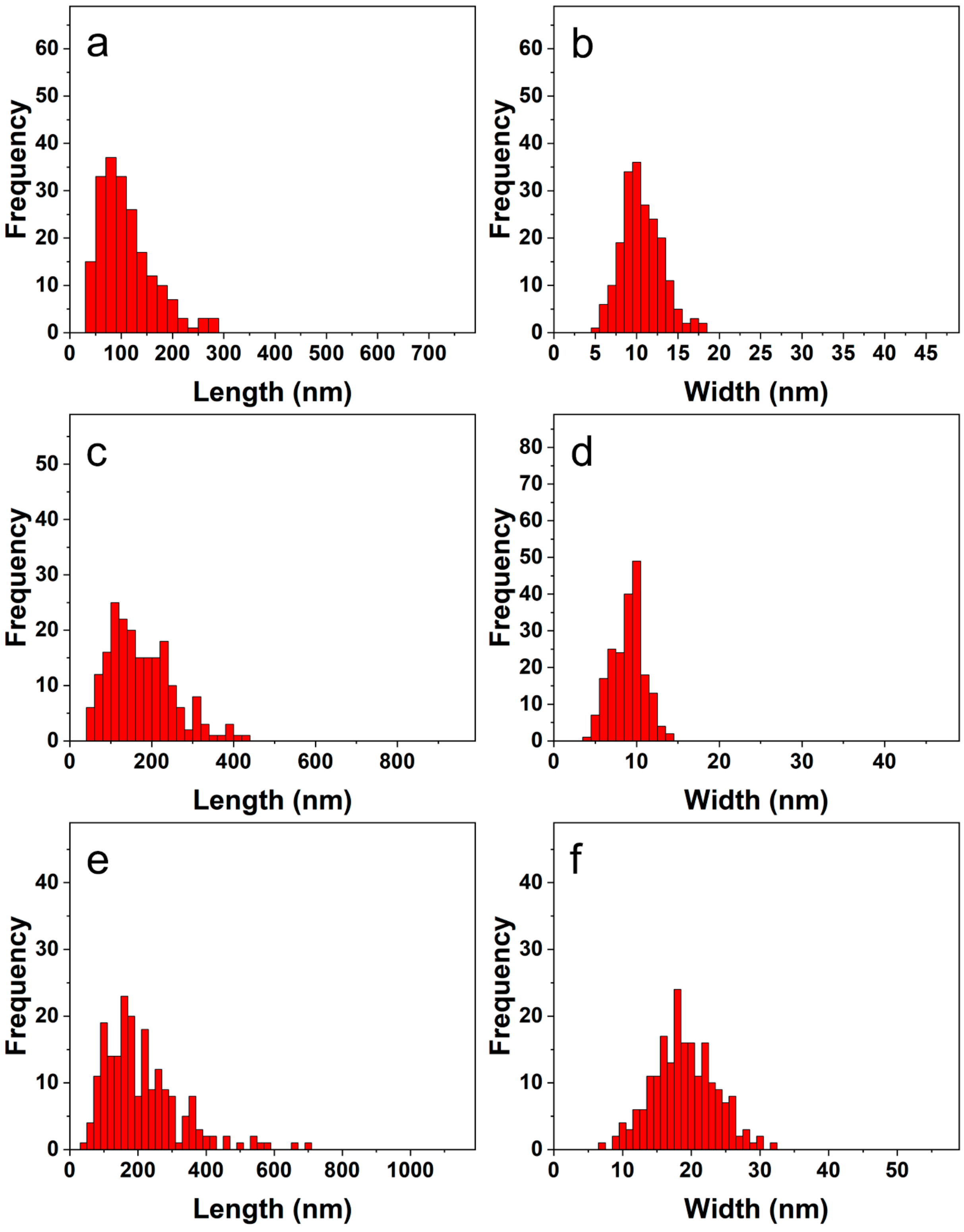

Figure S26. The statistical distributions of the nanostructures formed by the PBI in aqueous solutions (c= 0.1 mg·mL^{-1}) underwent an ionization-neutralization process, (a) the length and (b) width of the 2D ribbon-like nanostructures formed by PBI-L, (c) the length and (d) width of the 2D ribbon-like nanostructures formed by PBI-D, (e) the length and (f) width of the 2D ribbon-like nanostructures formed by PBI-β.

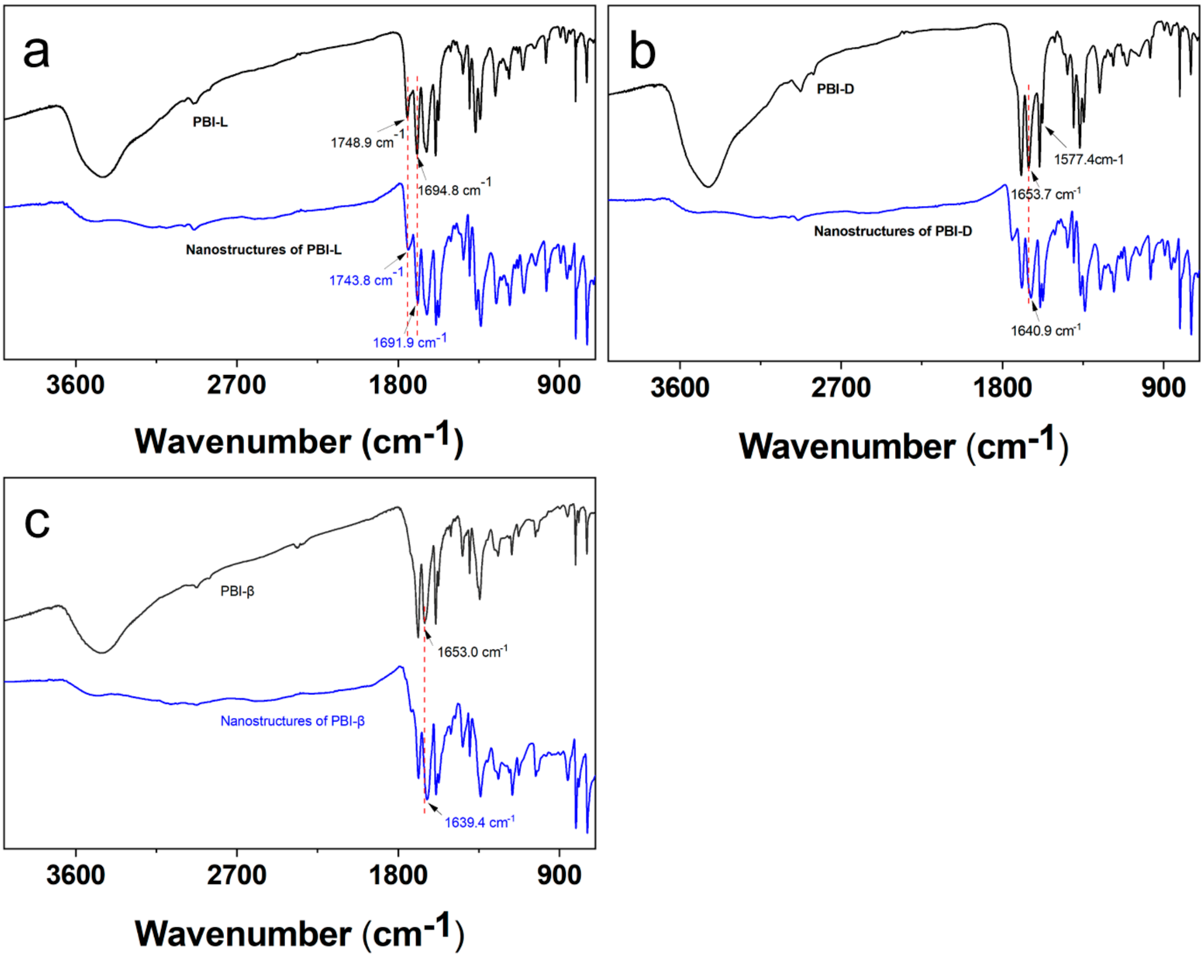

Figure S27. The FT-IR spectra of the 2D ribbon-like nanostructures formed by the PBI in the aqueous solutions (c= 0.1 mg·mL^{-1}) underwent an ionization-neutralization process, (a) PBI-L, (b) PBI-D, (c) PBI-β.

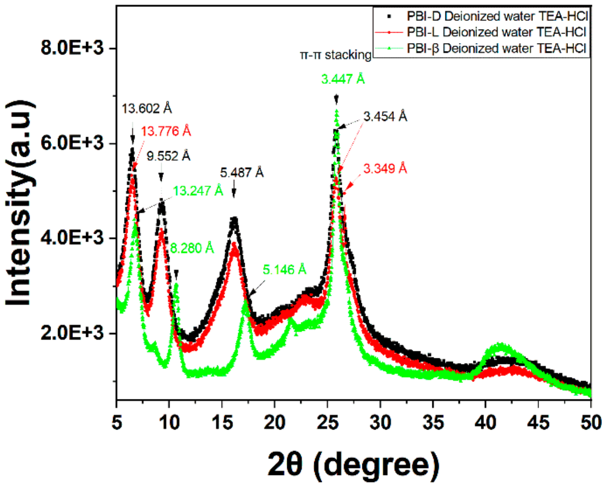

Figure S28. The XRD spectra of the 2D ribbon-like nanostructures formed by the PBI in the aqueous solutions (c= 0.1 mg·mL^{-1}) underwent a ionization-neutralization process.

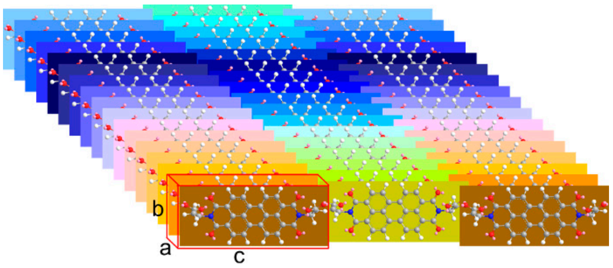

Figure S29. The probable unit cell of the 2D ribbon-like nanostructures formed by the PBI in the aqueous solutions (c= 0.1 mg·mL^{-1}) underwent a ionization-neutralization process.

Table S5. Summary of the XRD data of the nanostructures formed through an ionization-neutralization method

PBI	a (Å)		b (Å)	c (Å)
	a_1 (Å)	a_2 (Å)		
PBI-L	3.454	3.349	5.487	13.776
PBI-D	3.454		5.487	13.602
PBI-β	3.447		5.146	13.247

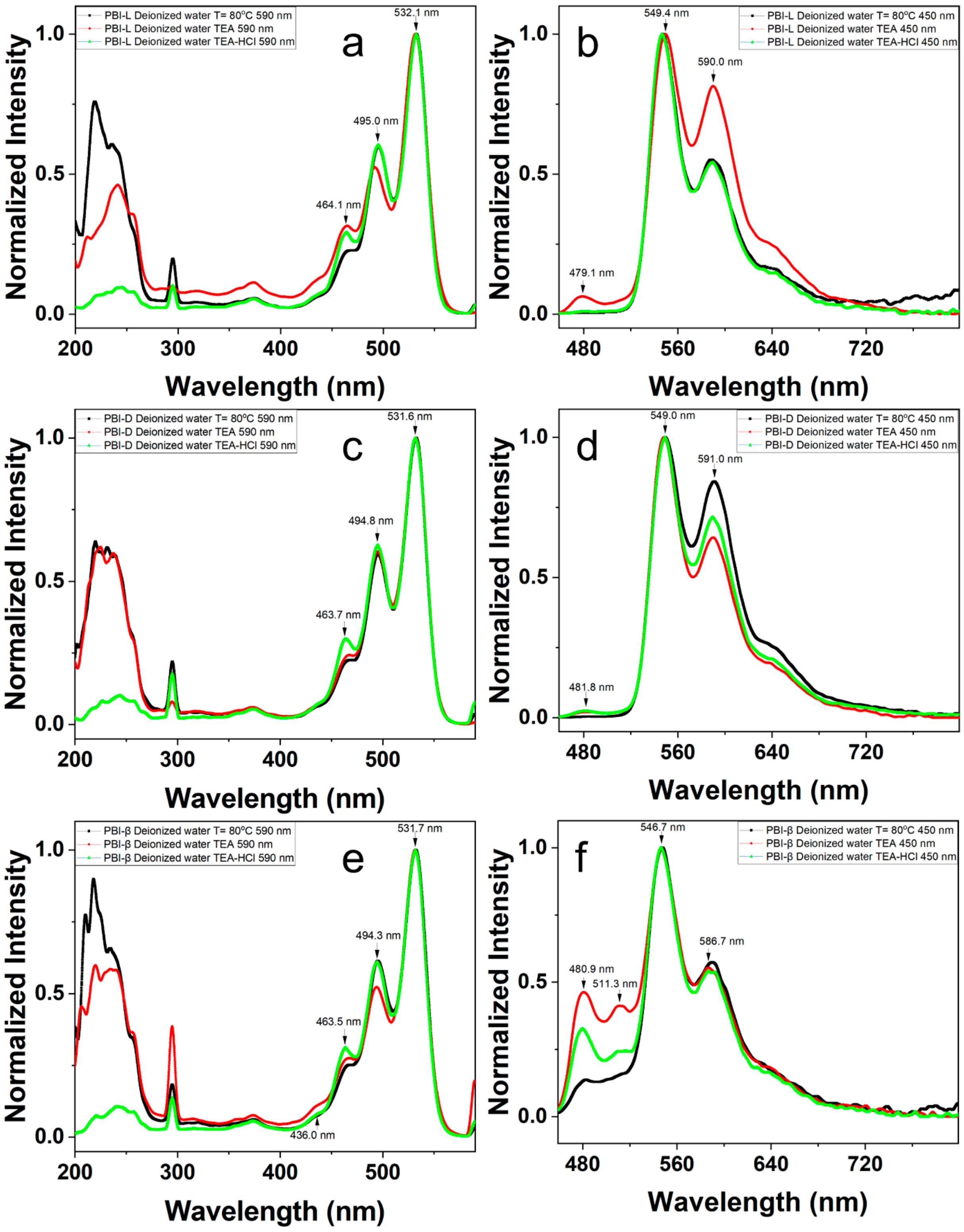

Figure S30. The fluorescence excitation spectra (at an emission band of 590 nm) (a, c, e) and the fluorescence emission spectra (excited by 450 nm) (b, d, f) of the PBI-L (a, b), PBI-D (c, d) and PBI-β (e, f) in aqueous solutions through different processes.
